# Half a Century of Temperate Non‐Forest Vegetation Changes: No Net Loss in Species Richness, but Considerable Shifts in Taxonomic and Functional Composition

**DOI:** 10.1111/gcb.70030

**Published:** 2025-01-24

**Authors:** Klára Klinkovská, Marta Gaia Sperandii, Ilona Knollová, Jiří Danihelka, Michal Hájek, Petra Hájková, Zdenka Hroudová, Martin Jiroušek, Jan Lepš, Jana Navrátilová, Tomáš Peterka, Petr Petřík, Karel Prach, Klára Řehounková, Jaroslav Rohel, Vojtěch Sobotka, Michal Vávra, Helge Bruelheide, Milan Chytrý

**Affiliations:** ^1^ Department of Botany and Zoology, Faculty of Science Masaryk University Brno Czech Republic; ^2^ Institute of Botany, Czech Academy of Sciences Průhonice Czech Republic; ^3^ Department of Paleoecology Institute of Botany, Czech Academy of Sciences Brno Czech Republic; ^4^ Department of Plant Biology Mendel University in Brno Brno Czech Republic; ^5^ Department of Botany, Faculty of Science University of South Bohemia České Budějovice Czech Republic; ^6^ Institute of Entomology Biology Centre of the Czech Academy of Sciences České Budějovice Czech Republic; ^7^ Experimental Garden and Gene Pool Collections Třeboň, Institute of Botany Czech Academy of Sciences Třeboň Czech Republic; ^8^ Faculty of Environmental Sciences Czech University of Life Sciences Prague Czech Republic; ^9^ Department of Vegetation Ecology, Institute of Botany Czech Academy of Sciences Průhonice Czech Republic; ^10^ Department of Biology, Faculty of Science University of Hradec Králové Hradec Králové Czech Republic; ^11^ Institute of Biology/Geobotany and Botanical Garden Martin Luther University Halle‐Wittenberg Halle Germany; ^12^ German Centre for Integrative Biodiversity Research (iDiv) Halle‐Jena‐Leipzig Leipzig Germany

**Keywords:** biodiversity change, drought, eutrophication, functional traits, habitat specialists, mesophilization, succession, vascular plants

## Abstract

In recent decades, global change and local anthropogenic pressures have severely affected natural ecosystems and their biodiversity. Although disentangling the effects of these factors is difficult, they are reflected in changes in the functional composition of plant communities. We present a comprehensive, large‐scale analysis of long‐term changes in plant communities of various non‐forest habitat types in the Czech Republic based on 1154 vegetation‐plot time series from 53 resurvey studies comprising 3909 vegetation‐plot records. We focused not only on taxonomic diversity but also on the functional characteristics of communities. Species richness of most habitat types increased over time, and taxonomic and functional community composition shifted significantly. Habitat specialists and threatened species became less represented in plant communities, indicating a decline in habitat quality. The spread of trees, shrubs, tall herbaceous plants, strong competitors, and nutrient‐demanding species in all non‐forest habitats, coupled with the decline of light‐demanding species, suggests an effect of eutrophication and natural succession following the abandonment of traditional management. Moreover, we identified specific trends in certain habitats. In wetlands, springs, and mires, moisture‐demanding species decreased, probably due to drainage, river regulations, and increasing drought resulting from climate change. Dry grasslands, ruderal, weed, sand, and shallow‐soil vegetation became more mesic, and successional processes were most pronounced in these communities, suggesting a stronger effect of abandonment of traditional management and eutrophication. In alpine and subalpine vegetation, meadows and mesic pastures, and heathlands, insect‐pollinated species declined, and the proportion of grasses increased. Overall, these functional changes provide deep insights into the underlying drivers and help conservationists take appropriate countermeasures.

## Graphical abstract

We present a comprehensive, large‐scale analysis of long‐term changes in plant communities of various non‐forest habitat types in the Czech Republic based on repeatedly sampled vegetation plots. Species richness of most habitat types increased over time, and taxonomic and functional community composition shifted significantly. The changes in the functional composition of plant communities indicated a decline in habitat quality caused by eutrophication and the abandonment of traditional management. Wetlands, springs, and mires showed signs of drying out, whereas dry habitats became more mesic.

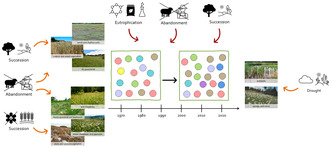

## 1 Introduction

Global change and increasing local anthropogenic pressures have seriously impacted natural ecosystems and their biodiversity in recent decades. The loss of species richness is well documented at the global scale (Barnosky et al. 2011; Ceballos et al. 2015; Pimm et al. 2014) but not always reflected in studies at regional and local scales (Bernhardt‐Römermann et al. 2015; Dornelas et al. 2014; Vellend et al. 2013). However, changes in species richness are not the only facet of biodiversity change. Large turnover in taxonomic community composition has been identified (Blowes et al. 2019; Hillebrand et al. 2018; Sperandii et al. 2021), and an important but often neglected aspect in studies of biodiversity change is a shift in functional community composition.

The functional characteristics of individual plant species in a community are determined by environmental conditions (Lavorel and Garnier 2002). Therefore, changes in the functional community composition can also indicate changes in environmental conditions. For instance, an increase in thermophilous plants would reflect the effects of climate change, and an increase in woody species would indicate a lack of disturbance, for example, after the abandonment of traditional management practices. Studies of the direct impact of environmental factors on plant communities are rare and challenging to conduct, as time‐series data often lack information on environmental factors, in particular at the fine scale that affects community composition. To assess their effects, changes in functional community characteristics can be used as proxies for biodiversity change drivers (Pakeman et al. 2009, 2017).

Although multiple factors contribute to biodiversity changes globally, the main threats differ among ecosystems and habitat types (Chytrý et al. 2019; Gigante et al. 2018; Janssen et al. 2016; Perzanowska and Korzeniak 2020). The response to different drivers may vary among habitat types depending on their characteristics (Smith, Knapp, and Collins 2009). Some drivers may affect certain habitats but not others. Therefore, habitat‐specific analyses are needed to identify which factors are responsible for the changes in a particular community.

Monitoring in permanent plots and repeated surveys of vegetation plots are commonly used methods to assess changes in plant communities (Chytrý et al. 2014; Kapfer et al. 2017). Repeated records of plant species composition at the same site represent the most precise tool to detect not only changes in species richness but also changes in species abundance, species composition, and functional community characteristics. Moreover, large databases collecting plot data from repeated vegetation surveys (Jandt, Bruelheide, Berg et al. 2022; Knollová et al. 2024; Pauli et al. 2015; Sperandii et al. 2022; Verheyen et al. 2017) and plant trait databases (Kattge et al. 2020; Kleyer et al. 2008; Klotz, Kühn, and Durka 2002; Weigelt, König, and Kreft 2020) have been compiled recently. These data facilitate synthetic studies of temporal changes in various biodiversity facets and their comparisons across large areas and multiple habitats.

We here explore patterns of long‐term changes in plant communities of various non‐forest habitats in the Czech Republic. This study aims to investigate changes at both community and species levels and focuses not only on changes in taxonomic diversity but also on functional and ecological characteristics in vegetation‐plot time series to gain insights into the underlying global and local factors. We hypothesize that (1) plant species diversity and functional composition of non‐forest habitats have changed over the last 50 years, reflecting changes in global and local environmental factors; (2) different habitat types underwent different changes triggered by different drivers; and (3) changes in functional community composition were more pronounced than changes in taxonomic diversity.

## 2 Materials and Methods

### 2.1 Study Area

The Czech Republic is a landlocked country in Central Europe (78,871 km^2^, 48.5° N–51° N, 12° E–19° E, elevation range 115–1603 m a.s.l.). The mean annual temperature in the Czech Republic was 7.4°C in 1971–1990 and 8.7°C in 2004–2023, with a maximum in July (16.8°C in 1971–1990 and 18.7°C in 2004–2023) and a minimum in January (−2.4°C in 1971–1990 and −1.1°C in 2004–2023; Czech Hydrometeorological Institute 2024a). The total annual precipitation was 655 mm in 1971–1990 and 679 mm in 2004–2023 (Czech Hydrometeorological Institute 2024b).

### 2.2 Data Acquisition

For the analysis of vegetation change in the non‐forest vegetation of the Czech Republic, we compiled a dataset of repeated vegetation‐plot records from the Czech Republic, which was then stored in the ReSurveyEurope database (Knollová et al. 2024). We selected plots sampled between 1971 and 2023 in plots of 1–100 m^2^ and plots without information on the plot size, which we considered to belong to this range, which is standardly used for sampling of non‐forest vegetation.

We classified our dataset into vegetation units (phytosociological syntaxa) using the classification expert system CzechVeg‐ESy (Chytrý et al. 2020) in the JUICE program (Tichý 2002). Then we selected vegetation‐plot time series that were, at the time of the first sampling, classified into one of the following broad habitats: alpine and subalpine vegetation; wetlands; springs and mires; wet meadows; mesic meadows and pastures; *Nardus* grasslands and heathlands; sand and shallow‐soil vegetation; dry grasslands; and ruderal and weed vegetation (see Appendix S1 for the list of alliances assigned to each broad habitat). We excluded vegetation‐plot time series that were undergoing rapid changes caused by local human influence, that is, plots established to track the successional development of vegetation, experimentally manipulated plots (e.g., experimentally grazed, mown or fertilized plots), and plots from recently restored sites. In the case of resurvey studies in which multiple resurvey plots were sampled to match each historical vegetation plot record, we used a conservative approach and selected only pairs of the most similar plot records according to Bray–Curtis dissimilarity in species composition to minimize pseudoturnover caused by relocation error (Verheyen et al. 2018).

In total, we obtained 1154 vegetation‐plot time series from 53 resurvey studies comprising 3909 vegetation‐plot records (Klinkovská et al. 2025). The length of the time series spanned from 3 to 48 years with a mean of 25. The plots were surveyed between 2 and 29 times, and the mean interval between the two consecutive surveys was 9 years. Approximately 50% of the plots were permanently marked in the field, and almost 80% of the plots were established in protected areas. For detailed characteristics of the dataset, see Table 1 and Appendix S2.

**TABLE 1:** Characteristics of the dataset used for the analysis. For more details, see Appendix S2.

	No. of time series	Time series length (years)			Interval length (years)			No. of surveys in time			Prop. of permanent plots (%)	Prop. of plots in protected areas (%)
		Mean	Min	Max	Mean	Min	Max	Mean	Min	Max		
All habitats	1154	25	3	48	9	1	48	3.4	2	29	48	77
Alpine and subalpine vegetation	73	48	17	48	23	3	48	2.3	2	3	1	100
Wetland vegetation	79	23	3	48	10	1	29	3.2	2	7	23	47
Spring and mire vegetation	154	20	3	48	6	1	45	3.8	2	21	53	95
Wet meadows	219	16	3	48	4	1	36	4.2	2	29	80	74
Mesic meadows and pastures	85	18	5	48	5	1	37	4.2	2	20	54	57
*Nardus* grasslands and heathlands	75	35	7	48	18	1	46	2.4	2	6	27	97
Sand and shallow‐soil vegetation	63	25	4	33	13	1	33	2.8	2	28	13	65
Dry grasslands	364	27	5	48	13	1	37	3	2	28	48	76
Ruderal and weed vegetation	42	20	5	48	6	1	35	4	2	7	79	79

For the analysis, we used only records of vascular plants. We excluded bryophytes and lichens from the species lists, as they were not sampled consistently across different studies. We standardized the taxonomic concepts and nomenclature of vascular plant species according to Kaplan et al. (2019). Most subspecies were merged to the species level, and some species were merged into aggregates. For species records determined only to the genus level, we checked the source data, and if a species was determined at a lower taxonomic level in a different sampling event of the same plot, we related this record to the lower‐level taxon (e.g., if *Viola* species was present in one time, and *Viola hirta* in another time in the same plot, *Viola* species was considered to be also *Viola hirta*; for details, see Appendix S3). If more than one lower‐level taxon occurred in another survey of the same plot, we equally distributed the cover of the genus‐level record among the lower‐level taxa. To minimize pseudoturnover caused by the misidentification of taxa in some surveys of a specific plot, we merged species we suspected to be misidentified under the name used in the last survey within a given time series. The list of these changes is available in Appendix S4. Moreover, we excluded the vernal taxa *Anemone nemorosa* and *Cardamine pratensis* agg. from the plots in the resurvey project CZ_0019_042 because the surveys were conducted in slightly different phenological stages (Klinkovská et al. 2023).

We converted categories of different cover scales used to estimate species cover in vegetation plots to percentages representing the mean value of each interval. In some resurvey studies, different cover scales were used in the different surveys. In such cases, we converted the different cover scales into the least precise scale used in the time series (usually the nine‐grade Braun‐Blanquet scale to the seven‐grade Braun‐Blanquet scale; Westhoff and van Der Maarel (1978)).

Species characteristics used in the analysis were obtained from the Pladias Database of the Czech Flora and Vegetation (Chytrý et al. 2021). They included growth form (Dřevojan 2020), life strategy scores (Guo and Pierce (2019) following the method of Pierce et al. (2017)), height (Kaplan et al. 2019), leaf characteristics (E‐Vojtkó et al. 2020; Findurová 2018; Kleyer et al. 2008; Klotz and Kühn 2002), flower characteristics (Durka 2002), reproduction type (Chrtek 2018; Durka 2002), dispersal strategy (Sádlo et al. 2018), myrmecochory (Konečná, Štech, and Lepš 2018), symbiosis with nitrogen fixers (Blažek and Lepš 2016), trophic mode (Těšitel et al. 2016), taxon origin (Pyšek et al. 2022), Ellenberg‐type indicator values (Chytrý et al. 2018), indicator values for disturbance of the herb layer (Herben, Chytrý, and Klimešová 2016), ecological specialization index (Zelený and Chytrý 2019), indices of colonization ability (Prach et al. 2017), and Red List status (Grulich 2017) (see Appendix S5 for more details).

### 2.3 Data Analysis

#### 2.3.1 Changes in Species Diversity and Community Characteristics

For each plot record, we calculated the species richness, Pielou's index of evenness (Shannon index/log(species richness)), and the community‐weighted and unweighted means for each species characteristic. To test the changes in these variables, we fitted a linear model for each variable (species richness, Pielou's evenness, and community‐weighted and unweighted means) with time as a predictor within each time series. We excluded time series consisting of plots of different sizes for the calculations of trends in species richness and Pielou's evenness.

We tested the probability of detecting a positive trend using a generalized additive model with a restricted maximum likelihood method, assuming a binomial error distribution (positive trend = 1, negative trend = 0). We included spatial coordinates as a smoothing term based on spherical splines to account for spatial autocorrelation and the resurvey study identity (a group of related time series) as a random effect in the model to account for methodological differences between the different resurvey studies. We considered the trends derived from the time series surveyed more times more reliable. Therefore, we weighted the observations of change by the square root of the number of surveys within the time series. We calculated the results for the whole dataset and each habitat type separately by including habitat identity as a predictor.

We visualized the differences between temporal trends in different habitats using a principal component analysis of the estimated trends for community–unweighted means resulting from the generalized additive model. In this analysis, we used only species characteristics with a significant trend in at least one habitat type and estimates based on at least five observations of change.

Some studies of biodiversity change used an alternative approach consisting of dividing each time series into separate observations of change between two consecutive years (e.g., Jandt, Bruelheide, Jansen et al. 2022). We compared the analysis using this method with our approach and found similar results using both methods (see Appendix S6 for more details and an alternative analysis).

#### 2.3.2 Changes in Species Composition

We tested changes in the species composition over time for each habitat using a constrained ordination (distance‐based redundancy analysis) with square‐root transformation of species percentage covers and square root of Bray–Curtis dissimilarity. Time was included in the model as a constraining variable, and plot identity was treated as a covariate. We tested the significance of changes in species composition over time using a permutation test with 999 permutations of observations within plots.

#### 2.3.3 Changes at the Species Level

We tested the changes in the presence of each species using the generalized linear model with binomial error distribution for species presence/absence data with time as a predictor. To test the changes in species cover, we fitted a linear model for species cover with time as a predictor within each time series. Time series consisting of plots of different sizes were excluded from this analysis. We extracted the slope of the trend from the model and coded the increasing trends as 1 and negative trends as 0. Then, we tested the probability of detecting a positive trend in changes in species cover or species presence using a binomial test, as the number of observations for many species was insufficient to use a more complicated model accounting for spatial autocorrelation. Only species with significant trends based on at least 30 observations of change from more than 15 sites (grid cells of 3′ of latitude × 5′ of longitude, approximately 5.5 × 6 km) were reported. We analyzed the whole dataset and then each habitat type separately. Species identified only to the genus level were excluded from this analysis.

The data analysis was performed using the R program (R Core Team 2023) with the packages vegan (Oksanen et al. 2022) and mgcv (Wood 2011). Functions from the packages tidyverse (Wickham et al. 2019); readxl (Wickham, Bryan, et al. 2023); and broom (Robinson et al. 2023) were used for the data handling. Graphs were created using functions from the packages ggplot2 (Wickham 2009); patchwork (Pedersen 2024); and scales (Wickham, Pedersen, et al. 2023).

## 3 Results

### 3.1 Changes in Species Richness and Diversity Indices

**FIGURE 1:**
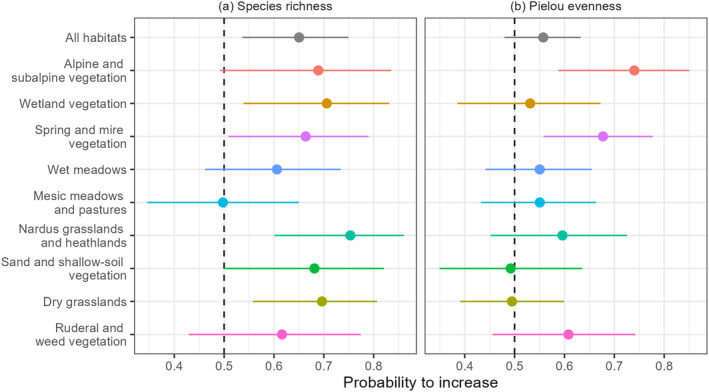
The probability of detecting a positive trend for (a) species richness (number of vascular plant species) and (b) Pielou's evenness in each habitat within each time series. Points represent the probability estimates from the generalized additive models, and lines represent the 95% confidence intervals.

We identified a few significant temporal changes in taxonomic diversity at the community level. There were significant positive trends for species richness in wetlands, springs and mires, *Nardus* grasslands and heathlands, and dry grasslands (Figure 1a). Positive trends for Pielou's evenness were detected in spring and mire, and alpine and subalpine vegetation (Figure 1b).

### 3.2 Changes in Species Composition and Individual Species

**FIGURE 2:**
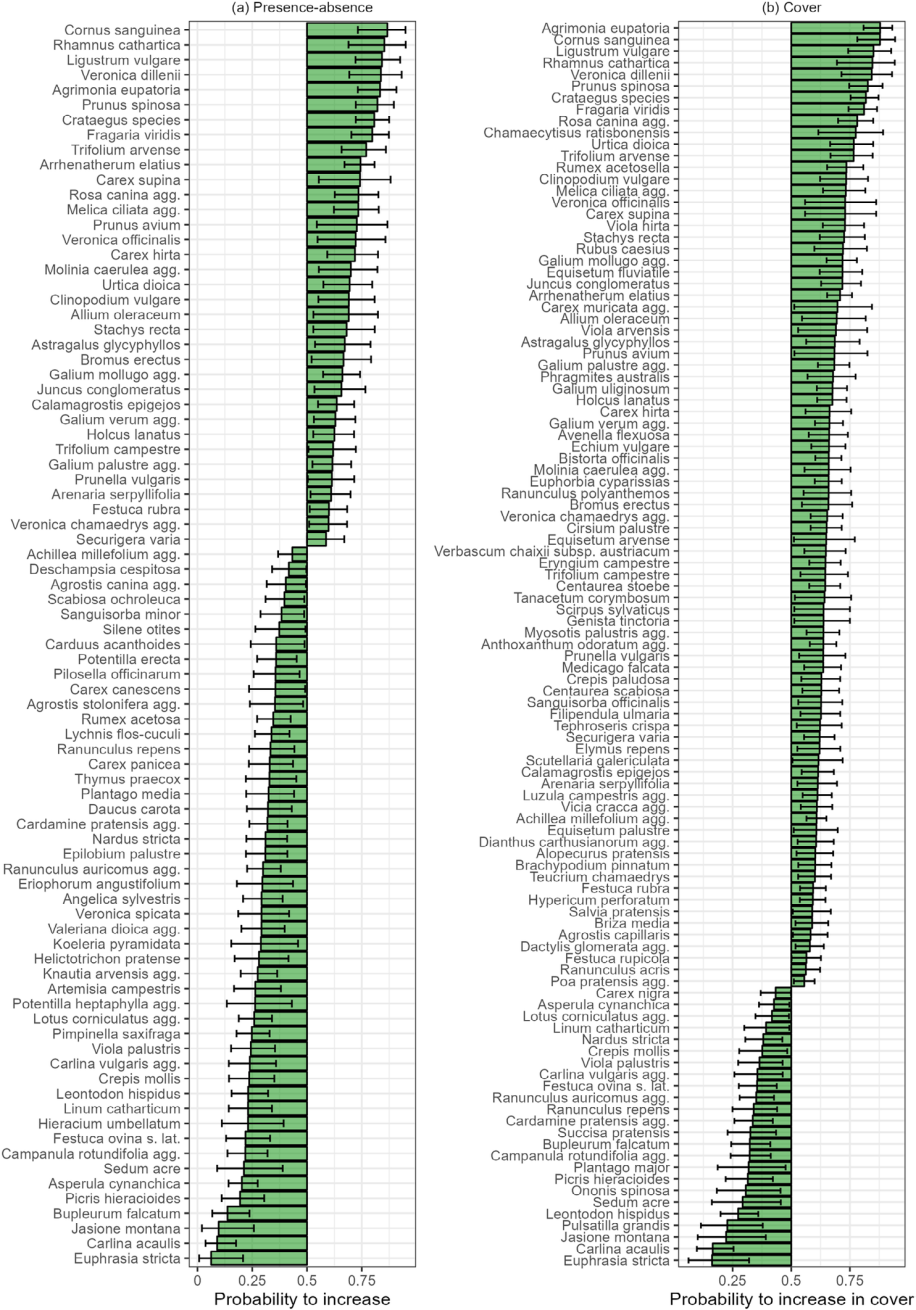
The probability of an increase in (a) presence and (b) cover of individual species. The green bars represent the estimated probability of increase from the binomial test, and the error bars indicate the 95% confidence intervals. Only species with significant trends according to the test based on at least 30 observations of change in more than 15 grid cells are shown. See Appendix S7 for the list of all species with significant positive and negative trends in the whole dataset and lists of species increasing or decreasing in different habitat types.

Although not many significant changes in plant taxonomic diversity at the plot level were detected, all habitats showed significant temporal changes in species composition (*p* < 0.001). Of the 1147 taxa included in the analysis, a significant decrease was found in the presence of 104 species and a significant increase in 109 species (Appendix S7). A significant decrease in cover occurred in 60 species, whereas the cover of 172 species significantly increased. Many shrubs (e.g., *Cornus sanguinea* , *Crataegus* spp., *Ligustrum vulgare* , *Prunus spinosa* , *Rhamnus cathartica* , and *Rosa canina* agg.) and competitive grasses (e.g., *Arrhenatherum elatius* , *Bromus erectus* , and *Calamagrostis epigejos* ) significantly increased in both their presence and cover (Figure 2). Specialists of dry grasslands and pastures (e.g., *Bupleurum falcatum*, *Carlina acaulis*, *Jasione montana* , and *Pulsatilla grandis*) but also specialists of springs and mires, wetlands, and wet meadows (e.g., *Eriophorum angustifolium* , *Valeriana dioica* agg., and *Viola palustris* ) or grasslands at higher altitudes (e.g., *Crepis mollis*) appeared among the decreasing species. Species significantly increasing or decreasing in each habitat type are listed in Appendix S7.

### 3.3 Common Patterns of Changes in Functional and Ecological Characteristics

**FIGURE 3:**
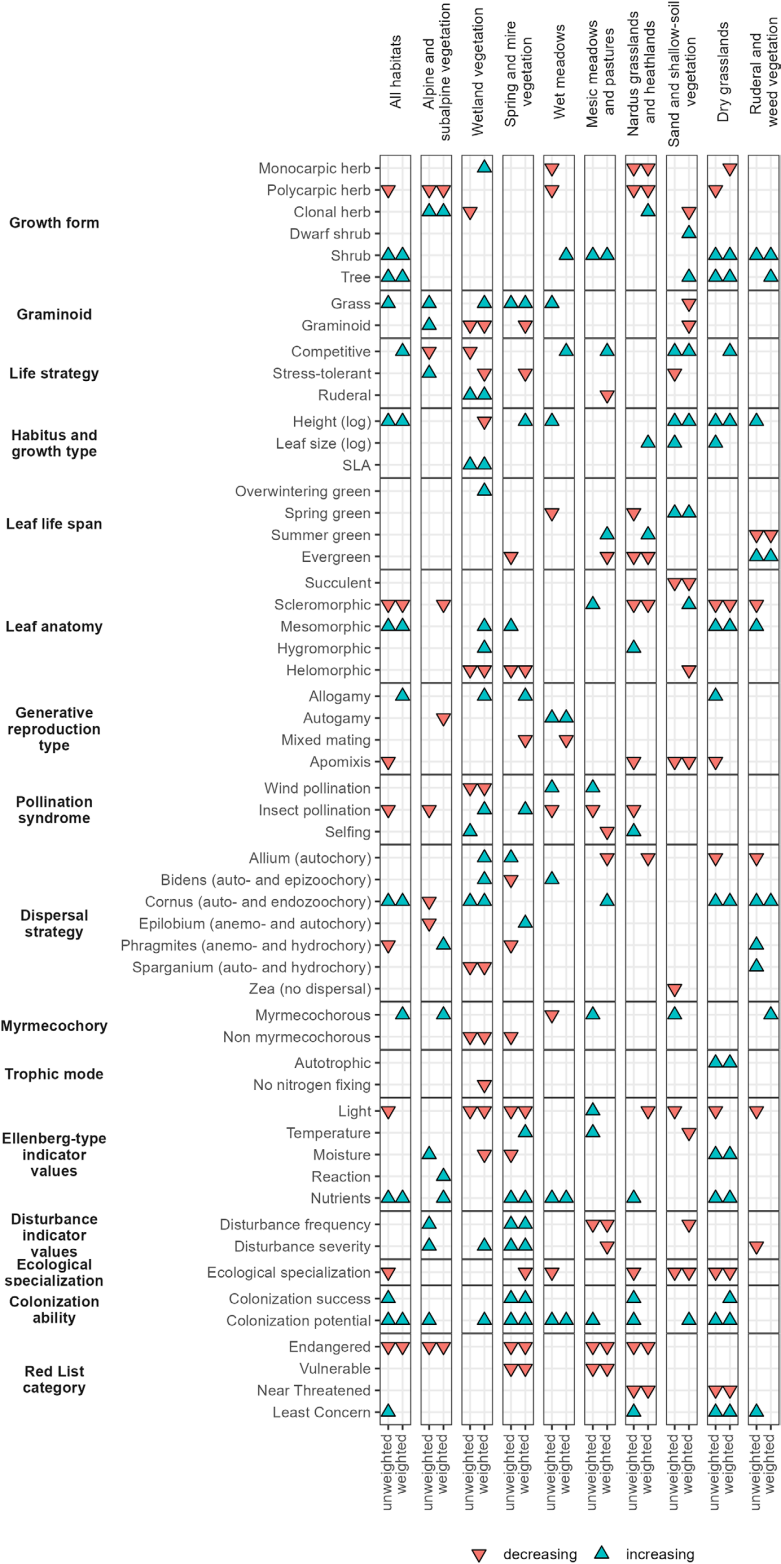
Significant trends in community‐unweighted and cover‐weighted means for each species characteristic and habitat. Only species characteristics with at least one significant trend are shown.

Across the whole dataset of the non‐forest vegetation, we identified a significant increase of trees, shrubs, tall plants, grasses, strong competitors, species with mesomorphic leaves, allogamous, autochorous, zoochorous, and myrmecochorous species, species with higher nutrient requirements, successful colonizers of new habitats and Least Concern species (Figure 3). In contrast, polycarpic herbs, species with scleromorphic leaves, apomictic, insect‐pollinated, anemochorous, hydrochorous, light‐demanding, more specialized, and threatened species became less represented in plant communities. There was no significant trend in the representation of neophytes. Compared with the results from the linear trend approach presented here, the interval change approach revealed fewer significant changes but of the same direction (Appendix S8).

### 3.4 Differences Between Habitat Types

**FIGURE 4:**
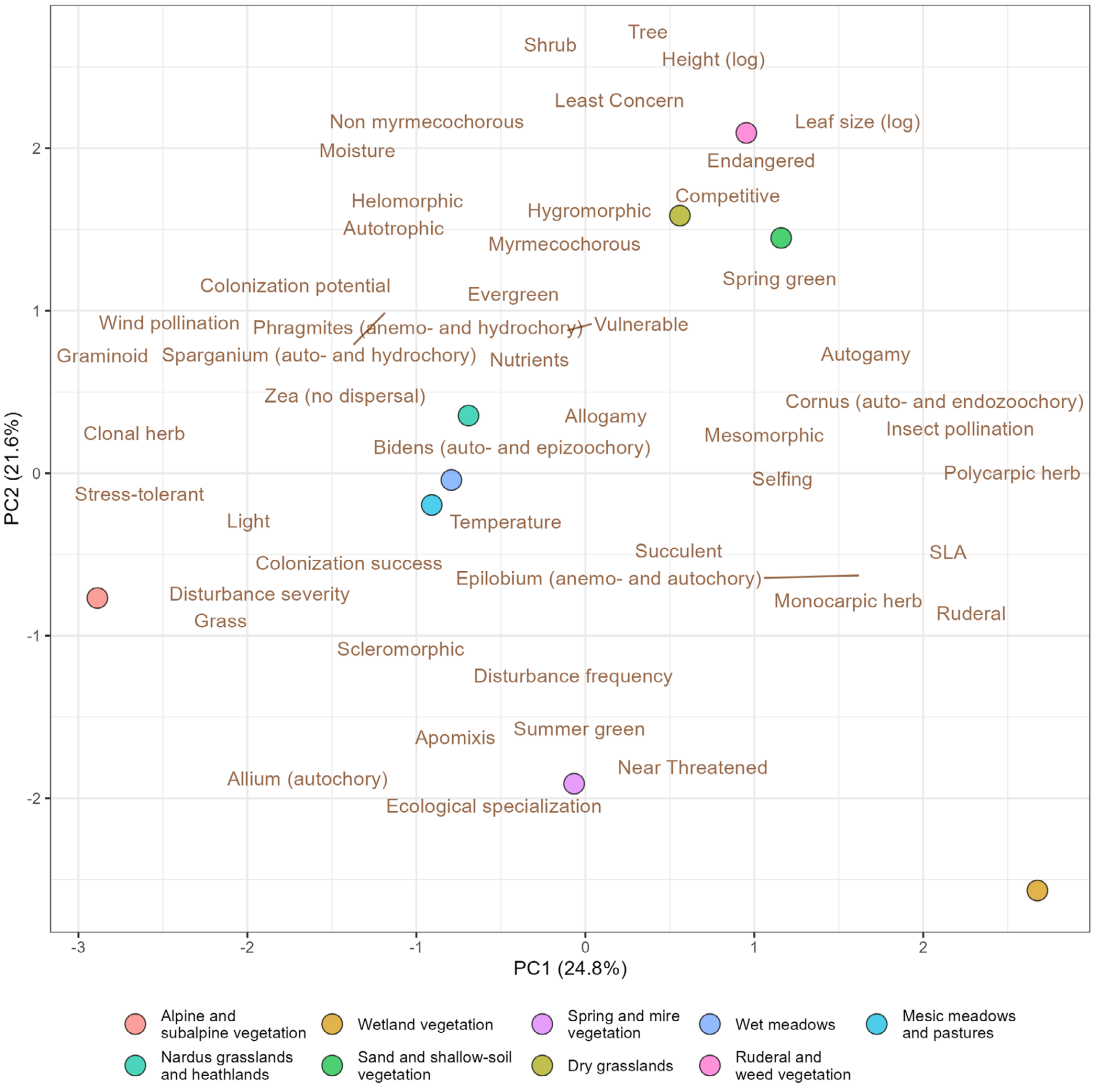
Differences in changes in community unweighted means of species characteristics between habitats. Only characteristics with at least one significant trend were included in the PCA.

There were considerable differences in temporal trends between habitats (Figure 4). Along the first ordination axis, wetlands and alpine and subalpine vegetation were clearly separated from the other habitats at the opposite ends of the gradient. In wetlands, ruderal species and species with higher SLA increased. In the alpine and subalpine vegetation, the proportion of grasses and graminoids increased the most, and there was a significant increase in clonal herbs, stress‐tolerant species and species adapted to stronger disturbances (Figure 3, Appendix S8). Insect‐pollinated species declined in the alpine and subalpine vegetation, meadows and mesic pastures, and *Nardus* grasslands and heathlands.

Species with higher moisture requirements decreased in wetlands, springs, and mires, whereas there was no or positive trend for moisture indicator values in the other habitats. This was also associated with the decrease of species with helomorphic leaves adapted to wet conditions in wetlands, springs, and mires. The representation of grasses significantly increased in these habitats, whereas the proportion of graminoids declined, indicating the decrease of specialized *Cyperaceae* species and *Juncus* for the benefit of grasses. In dry grasslands, ruderal and weed vegetation, and sand and shallow‐soil vegetation, displayed at the opposite end of the second ordination axis, the increase was most pronounced in competitively strong tall species with large leaves, trees, and shrubs. At the same time, species with higher requirements for light decreased most in these habitats. Wind‐pollinated species significantly increased in meadows and mesic pastures; however, they declined in wetlands.

## 4 Discussion

Based on the analysis of repeated vegetation‐plot records, we did not identify any significant negative trends in species richness and evenness in non‐forest plant communities in the Czech Republic. In most habitats, the species richness increased over time. However, there were nonrandom changes in community composition reflected in shifts in functional characteristics. Trees, shrubs, taller plants, stronger competitors, species successful in colonization of new habitats, and species with high nutrient requirements increased significantly in most non‐forest habitats. In contrast, insect‐pollinated, light‐demanding, highly specialized, and threatened species became less represented in plant communities. Moreover, we identified considerable differences in temporal trends between habitats. Moisture‐demanding species decreased in wetlands, springs, and mires, whereas mesophilous species increased in dry grasslands. The increase of taller species with larger leaves, competitively strong species, trees, and shrubs was most pronounced in dry grasslands, ruderal and weed vegetation, and sand and shallow‐soil vegetation, whereas mostly grasses increased in alpine and subalpine vegetation; wetlands; springs and mires; and wet meadows.

### 4.1 No Net Loss in Species Richness

Previous studies of temporal changes in species richness have shown contrasting results. Synthetic studies of community‐level species richness change over time (Bernhardt‐Römermann et al. 2015; e.g., Dornelas et al. 2014, 2019; Vellend et al. 2013) often show a balance between the number of decreasing and increasing species, whereas there is evidence for both increasing (Kolari et al. 2021; Navrátilová, Navrátil, and Hájek 2022; Steinbauer et al. 2018) and decreasing species richness (Harásek, Klinkovská, and Chytrý 2023; Klinkovská, Sperandii et al. 2024; Wesche et al. 2012) in different non‐forest habitats across Europe. We identified a significant increase in plot‐level species richness in most non‐forest habitats in the Czech Republic. However, the increase in species richness identified in most habitats should not be interpreted as an overall increase in the habitat quality as the increase in species richness might be caused by many different factors, including those leading to a habitat quality deterioration, e.g., an upward shift of species in the mountains due to climate warming (Lenoir et al. 2008; Pauli et al. 2012; Steinbauer et al. 2018; Walther, Beißner, and Burga 2005), expansions of generalist species and species typical for other habitats (Bergamini et al. 2009; Navrátilová, Navrátil, and Hájek 2022), or successional processes (Prach et al. 2014). These processes probably also contributed to the increases in species richness in our dataset because, at the same time, habitat specialists declined, and competitive and woody species became more abundant in the non‐forest vegetation. Moreover, the increase in species richness in time‐series data should be interpreted cautiously, as the positive trend is more likely to be detected because of the imbalance between the colonizations and extinctions under changing environmental conditions (Kuczynski, Ontiveros, and Hillebrand 2023) and because the list of species present in the plot is usually available for the resurvey study, and thus, the observer is more likely to detect more species than in the first survey.

### 4.2 Changes at the Species Level

We identified significant nonrandom turnover in the species composition of plant communities. Among the most increasing species, many woody species (e.g., *Cornus sanguinea* , *Crataegus* spp., *Ligustrum vulgare* , *Prunus spinosa* , and *Rosa canina* agg.) and competitive grasses (e.g., *Arrhenatherum elatius* , *Bromus erectus* , and *Calamagrostis epigejos* ) appeared. The spread of these species has also been documented in other European countries (Giarrizzo et al. 2017; Poniatowski et al. 2018; Ridding et al. 2020), and we confirmed the spread of several species assessed as expansive in the Czech Republic (Axmanová et al. 2024).

In line with studies of vegetation change in different parts of Europe (Bergamini et al. 2009; Diekmann et al. 2019; Klinkovská, Glaser et al. 2024; Peppler‐Lisbach et al. 2020), mainly habitat specialists (e.g., *Bupleurum falcatum*, *Carlina acaulis*, *Eriophorum angustifolium* , *Jasione montana* , *Pulsatilla grandis*, *Valeriana dioica* agg. and *Viola palustris* ) decreased significantly, which suggests a gradual decline in habitat quality.

### 4.3 Changes in Functional and Ecological Characteristics

Although the diversity of species functional traits belongs to the Essential Biodiversity Variables (Pereira et al. 2013) that should be considered when evaluating the state of biodiversity, the assessments of functional aspects are considerably less represented among studies of biodiversity change than studies of changes in community species richness. We quantified changes in functional community characteristics to identify possible causes of changes in plant communities. The increase in taller plants and stronger competitors, including woody species in all non‐forest habitats, together with the decrease in light‐demanding species (e.g., *Asperula cynanchica*, *Linum catharticum* , and *Koeleria pyramidata* ), suggests an effect of successional processes in the absence of appropriate management. This trend is consistent with the results of studies of changes in non‐forest vegetation across Europe (Finderup Nielsen, Sand‐Jensen, and Bruun 2021; Jandt, von Wehrden, and Bruelheide 2011; Pakeman et al. 2017; Smart et al. 2005) and may be attributed to the abandonment of traditional management of non‐forest habitats by grazing or mowing (Bauerkämper and Iordachi 2014; Bičík et al. 2015; Chytrý et al. 2017). The fact that these negative trends also appear in a dataset consisting of 80% of plots located in protected areas where conservation management has been introduced to maintain biodiversity raises concerns about the effectiveness of conservation management in the face of environmental changes.

The increase in indicator values for nutrients suggests that eutrophication caused by atmospheric nitrogen deposition, leaching and wind transport of nutrients from fertilizers applied to arable land is another driver contributing to changes in Czech plant communities. An increasing proportion of grasses in plant communities might also be interpreted as a result of eutrophication (Kirkham, Mountford, and Wilkins 1996; Smart et al. 2005). However, it is hard to separate the effects of eutrophication and natural succession because increased nutrient availability might also be caused by increased litter accumulation after the abandonment of traditional management (Enyedi, Ruprecht, and Deák 2008; Ruprecht et al. 2010), and successional processes might be fostered by an increased amount of nutrients in the ecosystem. Both processes support stronger competitors and likely contribute to the vegetation changes jointly (Diekmann et al. 2019; Jacquemyn, Brys, and Hermy 2003; Klinkovská, Sperandii et al. 2024). Moreover, increasing temperatures due to climate change might extend the growing season and thus foster biomass production and successional processes (Hillier, Sutton, and Grime 1994; Peringer et al. 2013; Sternberg et al. 1999; Wu et al. 2011).

Our results are in line with the national Red List classification (Grulich 2017), as we identified a significant decline in the proportion of threatened species in plant communities. The observed decline in ecologically specialized species (many of them included in the Red List) is consistent with the results of studies in different habitats across Europe (Diekmann et al. 2019; Finderup Nielsen, Sand‐Jensen, and Bruun 2021; Jansen et al. 2020; Klinkovská, Glaser, et al. 2024; Kolari et al. 2021; Peppler‐Lisbach et al. 2020). Ecologically specialized species might decline because of the spread of competitively strong species due to successional processes and eutrophication. Highly specialized species are often stress‐tolerant and adapted to nutrient‐poor conditions (Zelený and Chytrý 2019). The long‐term stability of such species depends on specific conditions (Hájek et al. 2020), they are often steadily outcompeted by stronger competitors supported by eutrophication or abandonment of traditional management (Czortek et al. 2018; Diekmann et al. 2014; Hautier, Niklaus, and Hector 2009; Krahulec et al. 2001; Louault et al. 2005). Although there is a general pattern of the decline of species with a narrow ecological niche, this species group consists of species adapted to different conditions, often near the extremes of environmental gradients (Zelený and Chytrý 2019). The factors contributing to a decline in habitat quality partly differ among habitats; therefore, it is necessary to identify the factors contributing to the decline of specialized species in particular habitats.

### 4.4 Differences Between Habitats

Changes in traits reflecting water availability differed considerably among habitat types. Whereas species with higher moisture demands decreased in wetlands, springs, and mires, species with higher Ellenberg‐type indicator values for moisture increased in alpine and subalpine vegetation and dry grasslands. Species with helomorphic leaves adapted to wet conditions declined in wetlands, springs, and mires, but at the same time, there was a significant decline of species with succulent and scleromorphic leaves adapted to dry conditions in *Nardus* grasslands and heathlands, sand and shallow soil vegetation, dry grasslands, and ruderal and weed vegetation. Furthermore, species with mesomorphic and hygromorphic leaves, which are in the middle of the leaf anatomy spectrum, increased in most habitat types.

The decline of species adapted to wet conditions in humid habitats such as springs, mires, and wetlands suggests not only a strong effect of local changes in water regime caused by drainage and river regulations but also increasing drought under climate change. Climate change effects are also indicated by the increase in species with higher Ellenberg‐type indicator values for temperature in springs and mires. Our results indicate that changes in hydrographic functioning belong to the most threatening factors for the wetland, spring, and mire vegetation, as suggested by the national Red List of habitats (Chytrý et al. 2019) and several resurvey studies across Europe (Koch and Jurasinski 2015; Mälson, Backéus, and Rydin 2008; Navrátilová, Navrátil, and Hájek 2022; Ortmann‐Ajkai et al. 2018; Pasquet, Pellerin, and Poulin 2015). The positive trend in evenness in springs and mires might be associated with increased herb layer productivity and abundance of grasses due to a decline in water level, as the decrease in water level leads to nutrient enrichment due to the mineralization of organic matter (Wassen and Olde Venterink 2006) and fosters successional processes by reducing anoxia. Competitively strong generalist grasses might also be supported by reduced disturbances after the abandonment of traditional management (Hájková et al. 2022). Competitive exclusion might then contribute to the decline of wetland, spring, and mire specialists. Although grasses significantly increased in these habitats, the representation of graminoids in wetlands and springs and mires declined, reflecting a decline of habitat specialists from the *Cyperaceae* family, for example, *Eleocharis palustris* agg. or *Eriophorum angustifolium* . The decline of these species is also reflected in the decreasing proportion of wind‐pollinated plants. Ruderal species increasing in wetlands (e.g., *Urtica dioica* ) might also be supported by drier and nutrient‐richer conditions.

Despite the increasing frequency of drought events and increased evapotranspiration under rising temperatures, mesophilous species increased in dry habitats. Together with the most pronounced spread of woody species and the decline of light‐demanding species in these habitats, this indicates a stronger effect of abandoning traditional management and consequent successional processes than global warming and drought events. For the competitively stronger mesophilous species, moisture might not be the limiting factor, they profit from the reduced disturbance regime, increased nutrient supply after the cessation of grazing or mowing, and longer growing season under climate change (Hájek et al. 2017; Jacquemyn, Brys, and Hermy 2003; Jernej et al. 2019; Kelemen et al. 2014; Louault et al. 2005; Wu et al. 2011). Moreover, the spread of mesophilous species can also be supported by eutrophication and increased atmospheric CO_2_ because less transpiration is needed to acquire the same amount of nutrients under increased nutrient levels or CO_2_ under increased atmospheric concentration, respectively; thus, less drought‐tolerant species might also survive in dry conditions (Kirschbaum and McMillan 2018; Swann et al. 2016; Walther 1960).

In line with studies from different European regions (Biesmeijer et al. 2006; Pakeman et al. 2017; Pan et al. 2024; Wesche et al. 2012), we identified a decline in insect‐pollinated plants, especially in alpine and subalpine vegetation, *Nardus* grasslands and heathlands, and meadows and pastures. This is probably another consequence of abandonment and eutrophication, which support the spread of competitive grasses over herbs (Batáry et al. 2010; Dupré et al. 2010; Ehlers, Bataillon, and Damgaard 2021).

### 4.5 Different Approaches to Detect a Temporal Trend

For the analysis of temporal trends in time‐series data, we preferred a linear trend approach that takes into account all data points from the given time series by fitting a linear trend over the whole time series. A similar approach was used, for example, by Dornelas et al. (2014, 2019). An alternative approach, interval change, was used, for example, by Jandt, Bruelheide, Jansen et al. (2022). It consists of dividing each time series into observations of change between two subsequent surveys. Trends detected by both approaches in our data mostly followed the same direction, but the linear trend approach detected more significant trends (Appendix S6). We consider the linear trend approach more appropriate for capturing long‐term trends in time‐series data because by dividing the time series into short intervals, short‐term fluctuations may be detected rather than long‐term trends. Such fluctuations can be caused by interannual dynamics due to weather fluctuations, which also exist in communities stable in the long term (Dostálek and Frantík 2011; Fischer et al. 2020). In contrast, directional long‐term trends might be masked by interannual fluctuations when both positive and negative short‐term changes occur, but one of them is of a larger magnitude. In such a case, there might be even an equal number of positive and negative interval changes but a considerable directional long‐term trend detectable by the linear trend approach.

### 4.6 Possible Drawbacks and Representativeness of the Results

Our dataset is not free of biases common in resurvey studies, such as relocation and observer error (Verheyen et al. 2018). However, we used a conservative approach and paid attention to minimizing pseudoturnover due to misidentification and relocation errors by thorough nomenclature unification and selection of the most similar pairs of plots in the case of resurvey studies in which multiple resurvey plots were sampled to match each historical plot. Especially, the trends in the species richness have to be interpreted with caution as these characteristics are more sensitive to observer and relocation biases (Verheyen et al. 2018) and changes in sampling effort in different surveys. However, according to Boch et al. (2019, 2022), detecting trends in functional traits and ecological characteristics is robust to these biases. Differences in sampling effort should affect the estimates of changes in these variables less than the changes in species richness, as the observer is likely to find more species when the list of species from the previous survey is available but would not intentionally look for specific species groups, such as species with higher indicator values for nutrients.

Our dataset also suffers from limited spatial representativeness and bias toward sites least affected by human influence. Permanent plots are typically established for vegetation monitoring, that is, at sites with the vegetation composition closest to the desired state where no big changes in management practices are expected. Resurvey studies are usually based on historical vegetation plots first sampled for vegetation classification purposes, for which the best‐developed communities of the given vegetation type were sampled. The most common practice while resampling historical vegetation plots is to sample vegetation with the most similar species composition as recorded originally. This conservative approach is also applied in the analyses when selecting the most similar new plot when several plots were resurveyed at the site of one historical plot. Moreover, sites that changed completely, for example, were transformed into arable land, afforested, or built up, were usually not resampled; thus, these large changes are not captured. Almost 80% of the plots included in our dataset were from protected areas, where conservation management has been introduced to maintain the habitat quality. Due to the preferential sampling, our results describe mainly trends in habitats of high quality at the time of the first sampling and at sites that experienced no major land‐use changes. Moreover, we did not consider changes in bryophytes, which can be more sensitive to environmental changes than vascular plants in some habitats, especially in springs and mires. Consequently, our results likely underestimate the overall trends of vegetation change in the Czech Republic.

## 5 Conclusions

The analysis of vegetation‐plot time series data showed considerable temporal changes in habitat quality of temperate non‐forest habitats across the Czech Republic. Species richness of most habitat types increased over time; however, changes in species composition and functional characteristics of plant communities revealed negative trends in habitat quality across all habitats. Among the declining species, insect‐pollinated, light‐demanding species, habitat specialists, and threatened species were more represented. On the other hand, competitively strong species, including woody species and grasses, increased together with nutrient‐demanding species, suggesting an effect of eutrophication and natural succession in the absence of appropriate conservation management. Differences in temporal trends between habitats suggest different importance of different threatening factors. The decline of moisture‐demanding species in wetlands, springs, and mires reflected changes in water regime that might have been caused by drainage, river regulations, and increasing drought under climate change. In dry grasslands, ruderal and weed vegetation, and sand and shallow soil vegetation, competitively strong species, trees, and shrubs increased most in these habitats suggesting a stronger effect of successional processes. Natural succession and eutrophication might also contribute to the changes in the alpine and subalpine vegetation, meadows and mesic pastures, *Nardus* grasslands and heathlands, where insect‐pollinated species declined the most and the representation of grasses increased.

## Author Contributions

**Klára Klinkovská:** conceptualization, data curation, formal analysis, methodology, resources, visualization, writing – original draft, writing – review and editing. **Marta Gaia Sperandii:** supervision, writing – review and editing. **Ilona Knollová:** data curation, resources. **Jiří Danihelka:** resources, writing – review and editing. **Michal Hájek:** resources, writing – review and editing. **Petra Hájková:** resources, writing – review and editing. **Zdenka Hroudová:** resources, writing – review and editing. **Martin Jiroušek:** resources, writing – review and editing. **Jan Lepš:** resources, writing – review and editing. **Jana Navrátilová:** resources. **Tomáš Peterka:** resources, writing – review and editing. **Petr Petřík:** resources, writing – review and editing. **Karel Prach:** resources, writing – review and editing. **Klára Řehounková:** resources, writing – review and editing. **Jaroslav Rohel:** resources, writing – review and editing. **Vojtěch Sobotka:** resources, writing – review and editing. **Michal Vávra:** resources, writing – review and editing. **Helge Bruelheide:** conceptualization, methodology, supervision, writing – review and editing. **Milan Chytrý:** conceptualization, resources, supervision, writing – review and editing.

## Conflicts of Interest

The authors declare no conflicts of interest.

## Supporting information


Appendix S1.

Appendix S2.

**Appendix S3**.
**Appendix S4**.
**Appendix S5**.
**Appendix S7**.

Appendix S6.

Appendix S8.

## Data Availability

The data and R code that support the findings of this study are openly available in Zenodo at https://doi.org/10.5281/zenodo.14589270 and GitHub at https://github.com/klaraklink/Half_a_century_of_temperate_non‐forest_vegetation_changes.git.
