# Supplementary material for: Half a Century of Temperate Non‐Forest Vegetation Changes: No Net Loss in Species Richness, but Considerable Shifts in Taxonomic and Functional Composition
Source: Glob Chang Biol. 2025 Jan 24;31(1):e70030. doi: 10.1111/gcb.70030 (PMC11758476; doi:10.1111/gcb.70030)
Supplement: Supplementary file 1 — Appendix S1. [file GCB-31-e70030-s004.docx]

**Supplementary information to the article:**

Klinkovská et al. Half a century of temperate non-forest vegetation changes: no net loss in species richness, but considerable shifts in taxonomic and functional composition.

**Appendix S1:** Phytosociological alliances belonging to broader vegetation types used for the analysis according to the national vegetation classification of the Czech Republic.

| Alpine and subalpine vegetation | Wetlands | Springs and mires | Wet meadows | Mesic meadows and pastures | Nardus grasslands and heathlands | Sand and shallow-soil vegetation | Dry grasslands | Ruderal and weed vegetation |
| --- | --- | --- | --- | --- | --- | --- | --- | --- |
| *Loiseleurio procumbentis-Vaccinion*  *Juncion trifidi*  *Nardo strictae-Caricion bigelowii*  *Agrostion alpinae*  *Calamagrostion villosae*  *Calamagrostion arundinaceae*  *Salicion silesiacae*  *Adenostylion alliariae*  *Dryopterido filicis-maris-Athyrion distentifolii* | *Eleocharition ovatae*  *Bidention tripartitae*  *Phragmition australis*  *Eleocharito palustris-Sagittarion sagittifoliae*  *Phalaridion arundinaceae*  *Glycerio-Sparganion*  *Carici-Rumicion hydrolapathi*  *Magno-Caricion elatae*  *Magno-Caricion gracilis* | *Caricion remotae*  *Swertio perennis-Dichodontion palustris*  *Caricion davallianae*  *Sphagno warnstorfii-Tomentypnion nitentis*  *Caricion canescenti-nigrae*  *Sphagno-Caricion canescentis*  *Sphagnion cuspidati*  *Sphagnion magellanici*  *Oxycocco palustris-Ericion tetralicis*  *Oxycocco microcarpi-Empetrion hermaphroditi* | *Molinion caeruleae*  *Deschampsion cespitosae*  *Calthion palustris* | *Arrhenatherion elatioris*  *Polygono bistortae-Trisetion flavescentis*  *Cynosurion cristati* | *Nardion strictae*  *Nardo strictae-Agrostion tenuis*  *Violion caninae*  *Euphorbio cyparissiae-Callunion vulgaris*  *Genisto pilosae-Vaccinion* | *Corynephorion canescentis*  *Armerion elongatae*  *Hyperico perforati-Scleranthion perennis*  *Arabidopsion thalianae*  *Alysso alyssoidis-Sedion*  *Festucion vaginatae* | *Alysso-Festucion pallentis*  *Diantho lumnitzeri-Seslerion*  *Festucion valesiacae*  *Cirsio-Brachypodion pinnati*  *Bromion erecti*  *Koelerio-Phleion phleoidis*  *Geranion sanguinei*  *Trifolion medii* | *Caucalidion*  *Veronico-Euphorbion*  *Oxalidion fontanae*  *Atriplicion*  *Malvion neglectae*  *Eragrostion cilianensi-minoris*  *Onopordion acanthii*  *Dauco carotae-Melilotion*  *Convolvulo arvensis-Elytrigion repentis*  *Aegopodion podagrariae*  *Fragarion vescae* |
