# Supplementary material for: Half a Century of Temperate Non‐Forest Vegetation Changes: No Net Loss in Species Richness, but Considerable Shifts in Taxonomic and Functional Composition
Source: Glob Chang Biol. 2025 Jan 24;31(1):e70030. doi: 10.1111/gcb.70030 (PMC11758476; doi:10.1111/gcb.70030)
Supplement: Supplementary file 2 — Appendix S2. [file GCB-31-e70030-s001.docx]

**Supplementary information to the article:**

Klinkovská et al. Half a century of temperate non-forest vegetation changes: no net loss in species richness, but considerable shifts in taxonomic and functional composition.

**Appendix S2:** Characteristics of the data used for the analysis.

*Table S2.1: Characteristics of the dataset used for the analysis.*

| **Code** | **Database name** | **Description** | **Study type** | **No. of time series** | **Survey year** | | **Time series length (years)** | | | **Interval length (years)** | | | **No. of surveys in time** | | |
| --- | --- | --- | --- | --- | --- | --- | --- | --- | --- | --- | --- | --- | --- | --- | --- |
|  |  |  |  |  | **start** | **end** | **mean** | **min** | **max** | **mean** | **min** | **max** | **mean** | **min** | **max** |
| CZ_0002 | CZ2_Rybnicek_Jizerky | Jizerské Mts (Rybníček´s plots) | resampling | 24 | 1991 | 2012 | 21 | 21 | 21 | 10 | 3 | 17 | 3 | 3 | 4 |
| CZ_0003 | CZ3_Rybnicek_Jeseniky + Czechia_NVD | Jeseníky Mts (Rybníček´s plots) | resampling | 25 | 1991 | 2008 | 15 | 14 | 17 | 15 | 14 | 17 | 2 | 2 | 2 |
| CZ_0004 | CZ4_Jemnice + Czechia_NVD | Rocky outcrops | permanent | 25 | 1993 | 2020 | 27 | 26 | 27 | 27 | 26 | 27 | 2 | 2 | 2 |
| CZ_0005 | CZ5_Kozenek | Kozének | permanent | 3 | 2015 | 2021 | 5 | 3 | 6 | 1 | 1 | 3 | 5 | 2 | 6 |
| CZ_0007 | CZ7_HuteGrasslandExp | PRHute_mowingexp | permanent | 5 | 2002 | 2023 | 21 | 21 | 21 | 1 | 1 | 2 | 21 | 21 | 21 |
| CZ_0008 | CZ8_ZavisinGrass_FertExp | FertilizationMedows | permanent (man) | 4 | 1990 | 2018 | 28 | 28 | 28 | 3 | 1 | 4 | 11 | 11 | 11 |
| CZ_0009 | CZ9_TrebonskoWetMead | Wet Meadows Trebon | resampling | 10 | 1984 | 2016 | 32 | 32 | 32 | 6 | 5 | 10 | 6 | 6 | 6 |
| CZ_0010 | CZ10_ChomoutovWetMead | Chomoutov wetland | permanent | 32 | 1998 | 2021 | 21 | 2 | 23 | 4 | 1 | 16 | 7 | 3 | 7 |
| CZ_0011 | CZ11_SWMoraviaAcidGrass | Acidic dry grasslands SW Moravia | resampling | 93 | 1986 | 2019 | 32 | 28 | 33 | 32 | 28 | 33 | 2 | 2 | 2 |
| CZ_0015 | CZ15_DevinDryGrass | Děvín dry grasslands | permanent | 7 | 1993 | 2021 | 28 | 28 | 28 | 1 | 1 | 2 | 28 | 28 | 28 |
| CZ_0016 | CZ16_ObranskaStran | Obřanská stráň | permanent | 11 | 1999 | 2021 | 22 | 22 | 22 | 7 | 1 | 20 | 4 | 4 | 4 |
| CZ_0018 | CZ18_EVLBoletice | Long-term monitoring EVL Boletice | permanent (man) | 9 | 2009 | 2023 | 14 | 12 | 14 | 1 | 1 | 1 | 15 | 13 | 15 |
| CZ_0019_003 | CZ19_003_CNFD_publishedsources_100122 | Abandoned wet grasslands with Carex brizoides - restoration experiment of former Calthion meadows | permanent (man) | 1 | 1993 | 2008 | 15 | 15 | 15 | 1 | 1 | 1 | 16 | 16 | 16 |
| CZ_0019_004 | CZ19_004_CNFD_publishedsources_101086 | Hodonínsko (Czech Republic), changes in sand vegetation in 5 years | permanent | 4 | 2002 | 2006 | 4 | 4 | 4 | 1 | 1 | 1 | 5 | 5 | 5 |
| CZ_0019_006 | CZ19_006_CNFD_publishedsources_101459 | Management experiment on wet meadows Rožnovská Bečva valley (Moravskoslezské Beskydy, Czech Republic) | permanent (man) | 6 | 2002 | 2019 | 17 | 17 | 17 | 4 | 1 | 10 | 5 | 5 | 5 |
| CZ_0019_008 | CZ19_008_CNFD_publishedsources_101473 | Monitoring of vegetation in Vokálova louka (Elbe river region, Central Bohemia) under constant management (mown once a year) | permanent | 1 | 1997 | 2016 | 19 | 19 | 19 | 1 | 1 | 1 | 20 | 20 | 20 |
| CZ_0019_009 | CZ19_009_CNFD_publishedsources_101474 | Fishpond, resampling of water vegetation | resampling | 1 | 2000 | 2015 | 15 | 15 | 15 | 8 | 4 | 11 | 3 | 3 | 3 |
| CZ_0019_010 | CZ19_010_CNFD_publishedsources_101537 | Fishpond, resampling of water vegetation | resampling | 1 | 1998 | 2015 | 17 | 17 | 17 | 9 | 7 | 10 | 3 | 3 | 3 |
| CZ_0019_011 | CZ19_011_CNFD_publishedsources_101539 | Vegetation changes in meadows of the basin of the Žebrákovský potok stream after 30 years | permanent | 19 | 1977 | 2005 | 25 | 6 | 28 | 14 | 6 | 27 | 3 | 2 | 3 |
| CZ_0019_014 | CZ19_014_CNFD_publishedsources_400833 | Change of vegetation in protected area Prokopské údolí in Prague | resampling | 9 | 1972 | 1994 | 22 | 21 | 22 | 20 | 9 | 22 | 2 | 2 | 3 |
| CZ_0019_015 | CZ19_015_CNFD_publishedsources_400840 | Change of vegetation in protected area Divoká Šárka in Prague | resampling | 6 | 1977 | 1993 | 15 | 14 | 16 | 10 | 5 | 16 | 3 | 2 | 3 |
| CZ_0019_018 | CZ19_018_CNFD_publishedsources_400871 | Monitoring of subxerophillous vegetation in protected area Prokopský vrch (Prague) | permanent | 3 | 1986 | 1996 | 10 | 10 | 10 | 1 | 1 | 4 | 8 | 8 | 8 |
| CZ_0019_021 | CZ19_021_CNFD_publishedsources_400879 | Resampling sandy vegetation with Jurinea cyanoides in 2 localities in the Czech Republic (protected area Písčina u Tišic and locality near Oleško) | resampling | 3 | 1983 | 1989 | 5 | 3 | 6 | 5 | 3 | 6 | 2 | 2 | 2 |
| CZ_0019_023 | CZ19_023_CNFD_publishedsources_101546 | Restoration of abandoned meadows with Carex brizoides to former Calthion meadows | permanent (man) | 3 | 1993 | 1998 | 4 | 4 | 5 | 1 | 1 | 2 | 5 | 4 | 6 |
| CZ_0019_024 | CZ19_024_CNFD_publishedsources_101547 | Changes of mire meadows in protected area Chvojnov affected by drainage in the 60s and restoration in 2013 | resampling | 6 | 2003 | 2019 | 10 | 6 | 16 | 7 | 6 | 10 | 2 | 2 | 3 |
| CZ_0019_025 | CZ19_025_CNFD_publishedsources_101548 | Long-term changes of alpine and subalpine vegetation of Králický Sněžník | resampling | 19 | 1971 | 2019 | 45 | 45 | 48 | 45 | 45 | 48 | 2 | 2 | 2 |
| CZ_0019_027 | CZ19_027_CNFD_publishedsources_400142 | Krkonoše Mts. | resampling | 17 | 1980 | 1995 | 14 | 11 | 15 | 14 | 11 | 15 | 2 | 2 | 2 |
| CZ_0019_030 | CZ19_030_CNFD_publishedsources_100125 | Changes in the steppe vegetation of the solitary conic hill Oblík in the České Středohoří Protected Landscape Area | permanent | 46 | 1972 | 2007 | 35 | 35 | 35 | 35 | 35 | 35 | 2 | 2 | 2 |
| CZ_0019_031 | CZ19_031_CNFD_publishedsources_100126 | NPR Oblík (České Středohoří Protected Landscape Area) | resampling | 18 | 1975 | 1992 | 6 | 2 | 17 | 2 | 1 | 10 | 3 | 2 | 5 |
| CZ_0019_033 | CZ19_033_CNFD_publishedsources_101014 | Influence of cement factory on vegetation | permanent | 2 | 1979 | 2010 | 31 | 31 | 31 | 6 | 2 | 15 | 6 | 6 | 6 |
| CZ_0019_035 | CZ19_035_CNFD_publishedsources_101409 | Changes in the vegetation of protected area Dolejší dráhy (district Klatovy, Czech Republic) | resampling | 4 | 2003 | 2012 | 9 | 9 | 9 | 9 | 9 | 9 | 2 | 2 | 2 |
| CZ_0019_036 | CZ19_036_CNFD_publishedsources_400660 | Change of ruderal community with Malva neglecta in part of Prague | resampling | 2 | 1975 | 1983 | 8 | 8 | 8 | 2 | 1 | 3 | 5 | 4 | 5 |
| CZ_0019_040 | CZ19_040_CNFD_publishedsources_401888 | Influence of afforestation of Pinus mugo on alpine vegetation in Krkonoše Mts (W part) | permanent | 9 | 1981 | 1998 | 5 | 3 | 17 | 4 | 3 | 8 | 2 | 2 | 4 |
| CZ_0019_042 | CZ19_042_Eastern_Sudetes_subalpine_vegetation | Jeseniky Mts (Czech Republic) - long-term change of grassland summit vegetation (abandonment, avalanche control) | resampling | 127 | 1973 | 2021 | 28 | 11 | 48 | 20 | 11 | 37 | 2 | 2 | 3 |
| CZ_0019_043 | CZ19_043_CNFD_publishedsources_101553 | Change of grassland vegetation in Hrnčířské louky protected area after 20 years | resampling | 55 | 1986 | 2006 | 19 | 18 | 20 | 19 | 18 | 20 | 2 | 2 | 2 |
| CZ_0019_044 | CZ19_044_CNFD_publishedsources_101436 | Change of vegetation in Křížky protected area in 10 years | permanent | 9 | 2004 | 2013 | 9 | 9 | 9 | 9 | 9 | 9 | 2 | 2 | 2 |
| CZ_0019_045 | CZ19_045_CNFD_publishedsources_101554 | Slavkovský les, change of grassland vegetation | permanent | 99 | 2005 | 2017 | 9 | 7 | 12 | 9 | 7 | 12 | 2 | 2 | 2 |
| CZ_0019_046 | CZ19_046_Straziste | Strážiště, change of grassland vegetation | resampling | 1 | 1994 | 2022 | 28 | 28 | 28 | 1 | 1 | 5 | 23 | 23 | 23 |
| CZ_0019_047 | CZ19_047_Smoravia_basiphilous_grass | Change of grassland vegetation in Central Moravian Carpathians | resampling | 90 | 1985 | 2022 | 37 | 37 | 37 | 37 | 37 | 37 | 2 | 2 | 2 |
| CZ_0019_049 | CZ19_049_CNFD_publishedsources_101584 | monitoring vegetation after hydrological restoration of peatland vegetation | permanent | 8 | 2017 | 2020 | 3 | 1 | 3 | 1 | 1 | 2 | 3 | 2 | 4 |
| CZ_0019_050 | CZ19_050_CNFD_publishedsources_101585 | monitoring grassland vegetation and influence of railway reconstruction | permanent | 10 | 1975 | 2006 | 31 | 31 | 31 | 9 | 1 | 30 | 4 | 3 | 5 |
| CZ_0019_051 | CZ19_051_Krtinsky_potok_valley | Křtinský potok valley | resampling | 7 | 2005 | 2022 | 15 | 13 | 17 | 15 | 13 | 17 | 2 | 2 | 2 |
| CZ_0019_054 | CZ19_054_old_field_succession_South_Moravia | Old field succession South Moravia | permanent | 75 | 2007 | 2023 | 15 | 14 | 16 | 15 | 14 | 16 | 2 | 2 | 2 |
| CZ_0021 | CZ21_EVLSumava | Šumava long-term monitoring | permanent (man) | 34 | 2014 | 2021 | 6 | 4 | 7 | 1 | 1 | 2 | 6 | 5 | 7 |
| CZ_0023 | CZ23_NPPodyji | NP-Podyji-Thayatal-Dry-Grasslands | resampling | 2 | 1991 | 2022 | 30 | 29 | 31 | 30 | 29 | 31 | 2 | 2 | 2 |
| CZ_0027 | CZ27_Ohrazeni | Ohrazeni | permanent (man) | 3 | 1994 | 2022 | 28 | 28 | 28 | 1 | 1 | 1 | 29 | 29 | 29 |
| CZ_0028 | CZ28_Milovice_LargeHerbiv | Milovice_grazing | permanent (man) | 35 | 2017 | 2022 | 5 | 4 | 5 | 1 | 1 | 3 | 5 | 4 | 5 |
| CZ_0029 | CZ29_NPPodyji_ElephantRidge | Podyji National Park, Elephant Ridge | resampling | 18 | 1996 | 2023 | 27 | 27 | 27 | 27 | 27 | 27 | 2 | 2 | 2 |
| CZ_0030 | CZ30_VysenskeKopce | NPR Vyšenske Kopce management | permanent | 15 | 2000 | 2022 | 13 | 2 | 22 | 2 | 1 | 9 | 9 | 3 | 14 |
| CZ_0031 | CZ31_TrebonFens_perm | TrebonFens_permanentplots | permanent | 30 | 2004 | 2019 | 15 | 15 | 15 | 15 | 15 | 15 | 2 | 2 | 2 |
| CZ_0080 | NA | Žďárské vrchy fens management experiment | permanent | 50 | 2007 | 2020 | 13 | 10 | 13 | 2 | 1 | 4 | 7 | 6 | 7 |
| CZ_0082 | NA | Sand vegetation South Moravia | resampling | 31 | 2003 | 2023 | 18 | 17 | 20 | 18 | 17 | 20 | 2 | 2 | 2 |
| CZ_0083 | NA | Polabí wetlands | resampling | 27 | 1991 | 2017 | 25 | 24 | 26 | 25 | 24 | 26 | 2 | 2 | 2 |


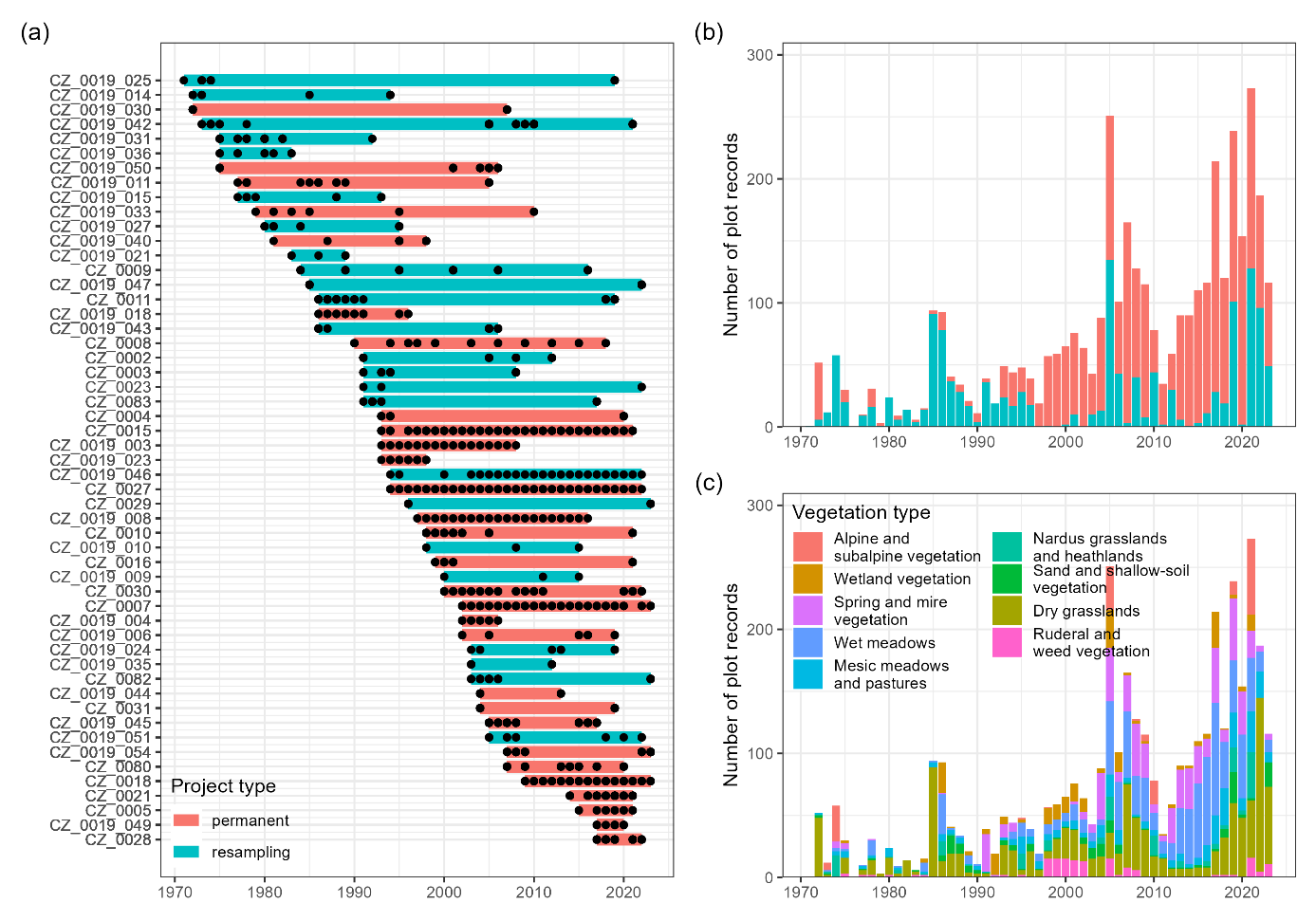


*Figure S2.1: Temporal distribution of the data. (a) the time period covered by each resurvey study, (b) and (c) a number of plot records in each year, (b)records of permanent and resurvey plots and (c) records of different vegetation types, are shown in different colours. Black points indicate sampling times.*


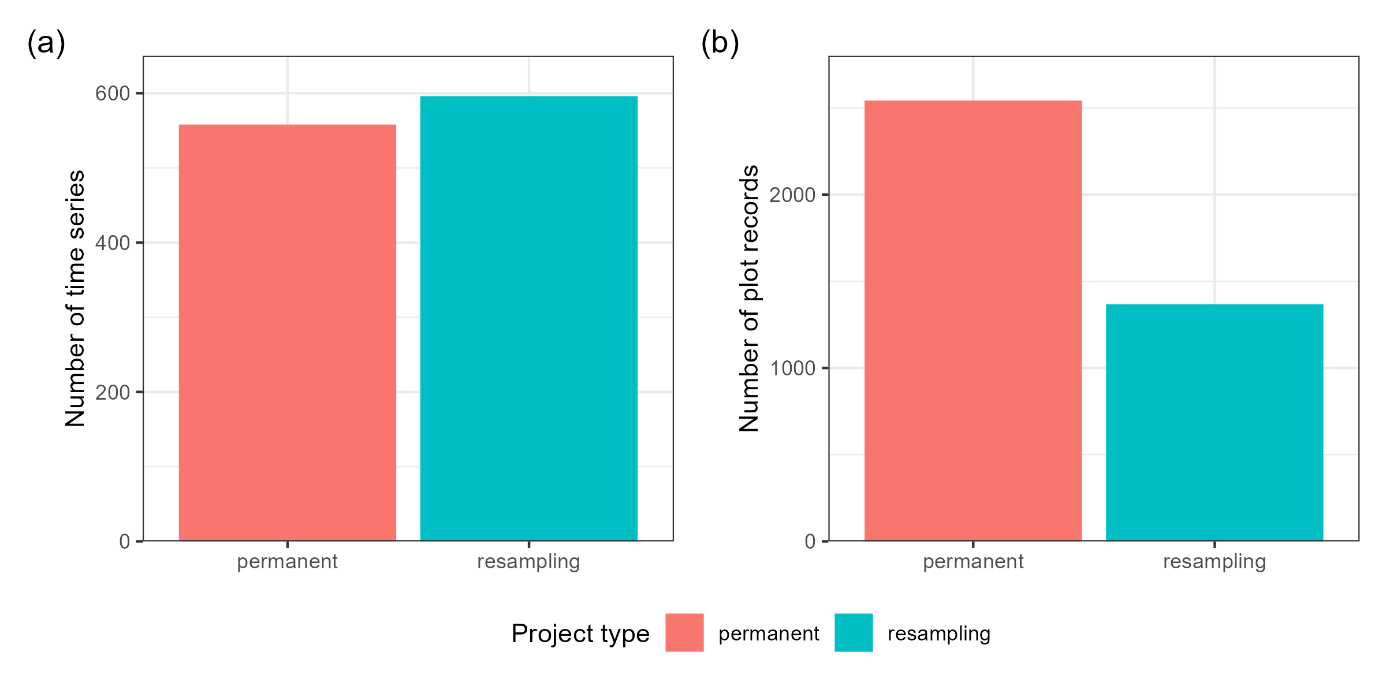


*Figure S2.2: Number of (a) vegetation-plot time series and (b) plot records with distinction of permanent and resurveyed plots.*


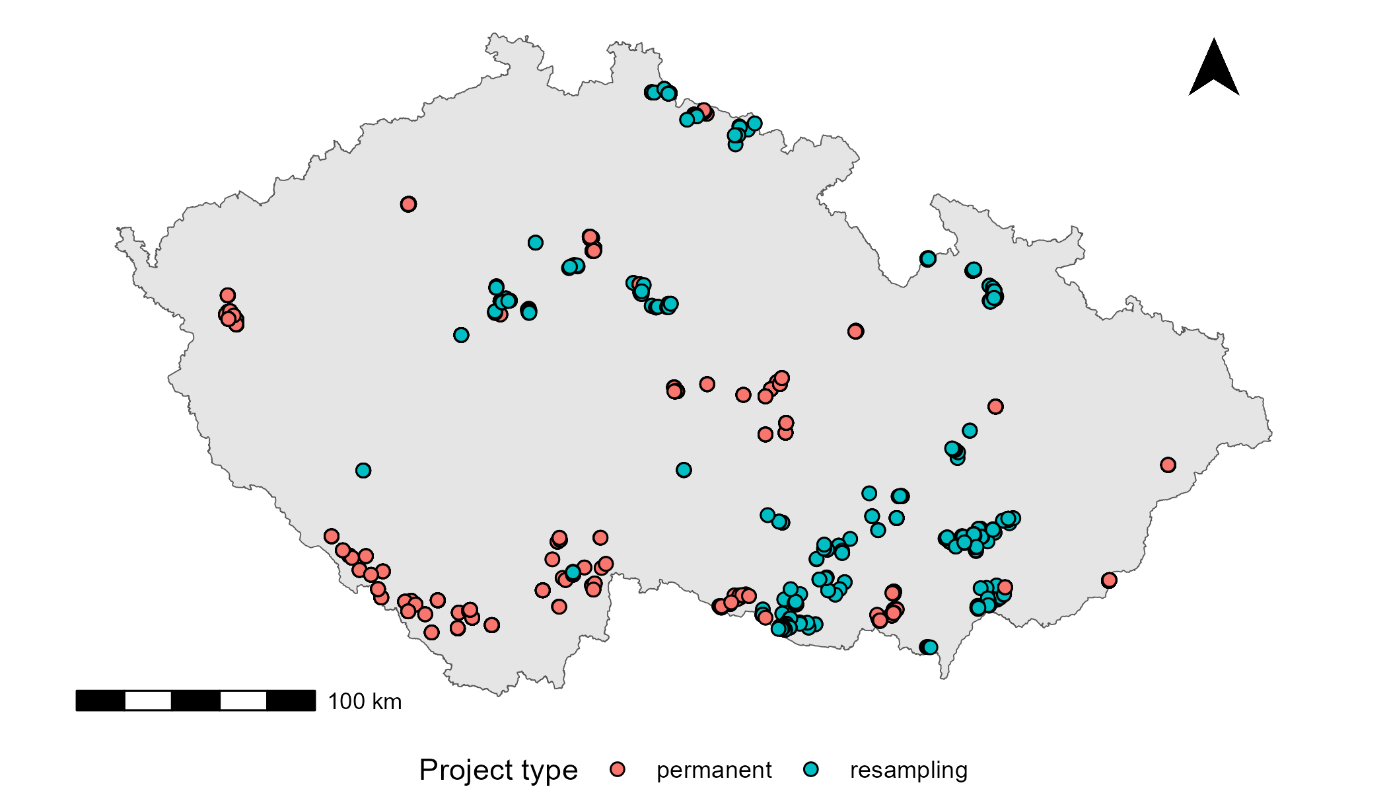


*Figure S2.3: Geographic distribution of the repeatedly sampled vegetation plots. Permanent and resurveyed plots are shown in different colours.*


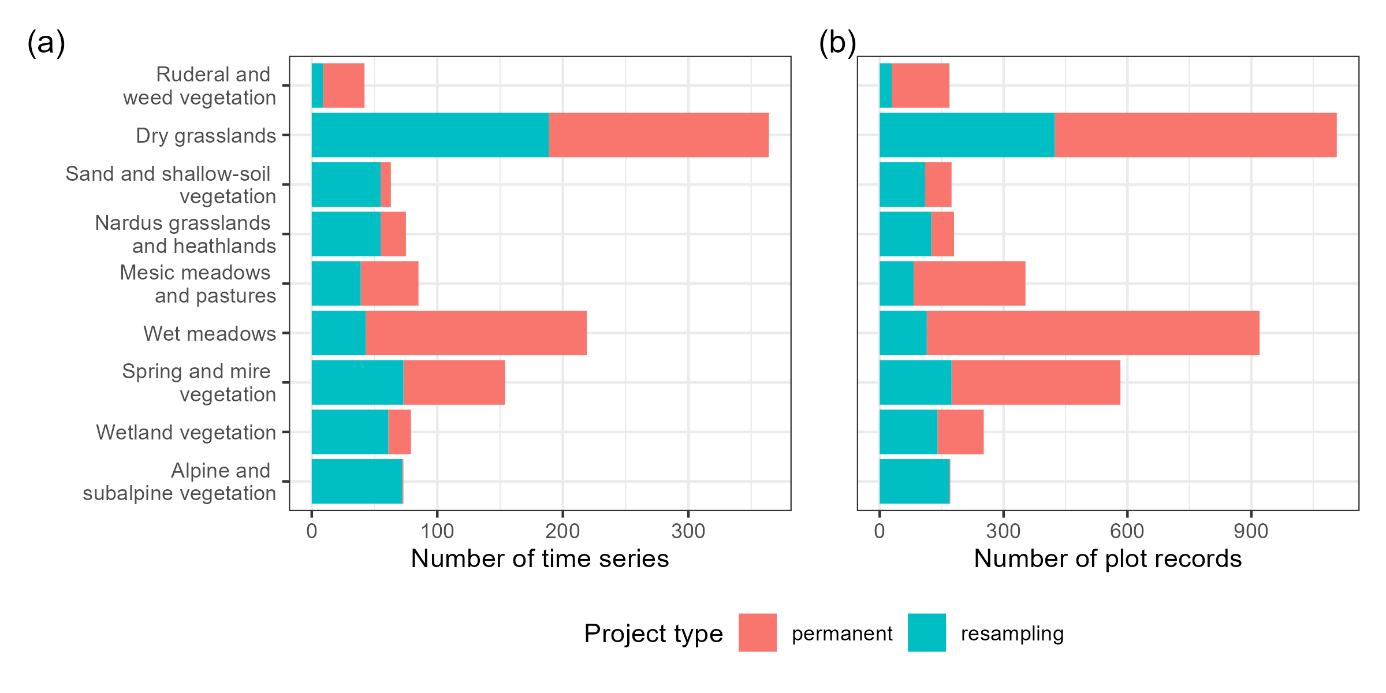


*Figure S2.4: Number of (a) vegetation-plot time series and (b) plot records of each habitat. Permanent and resurveyed plots are shown in different colours.*

*
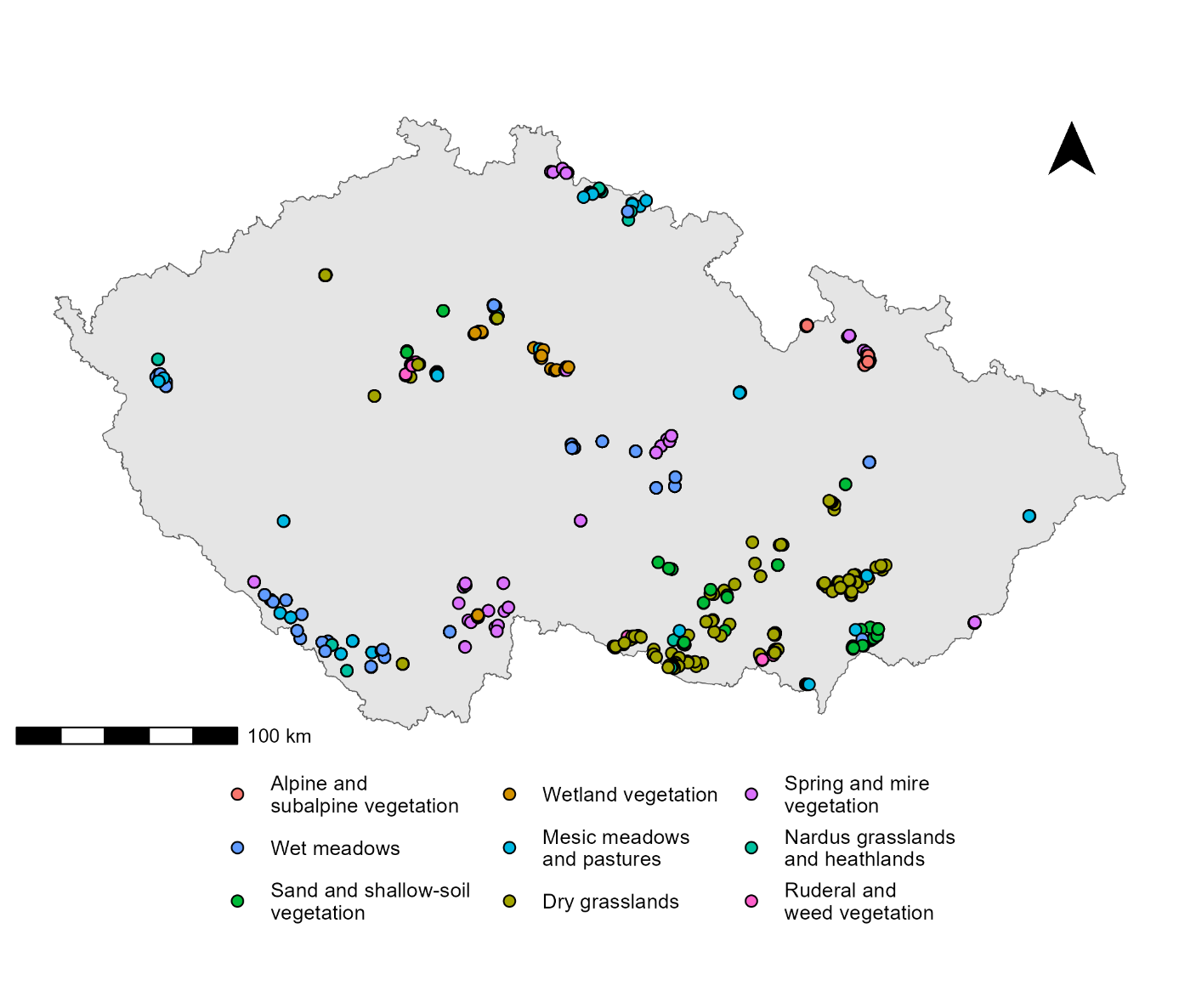
*

*Figure S2.5: Geographic distribution of repeatedly sampled vegetation plots. Plots of different habitats are shown in different colours.*


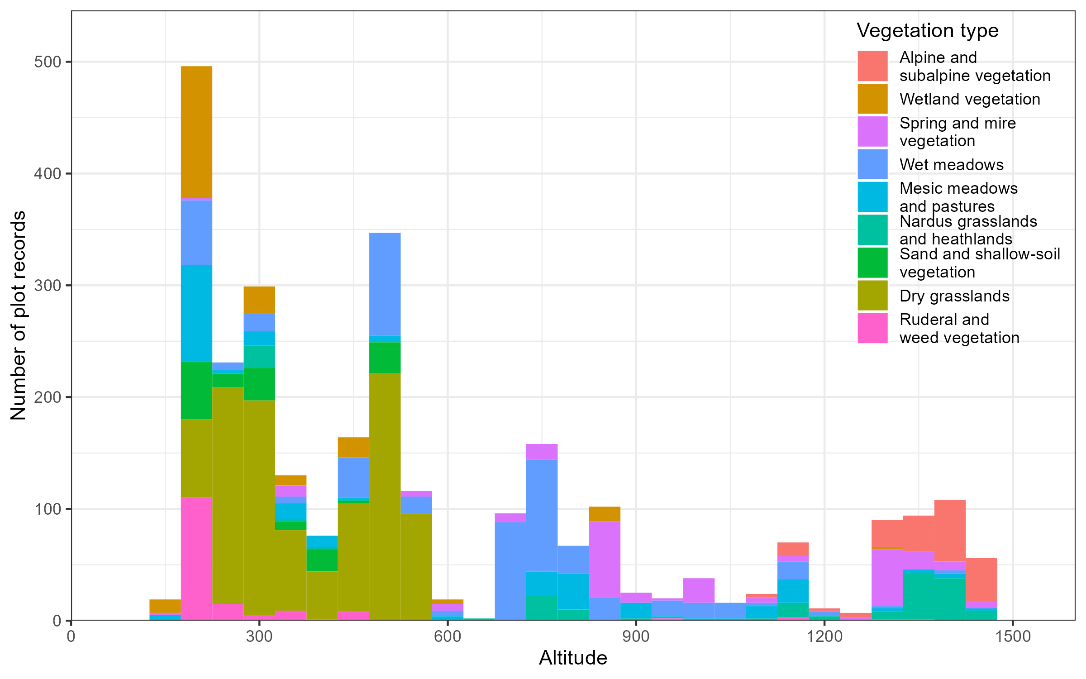


*Figure S2.6: Distribution of the data along an elevation gradient.*


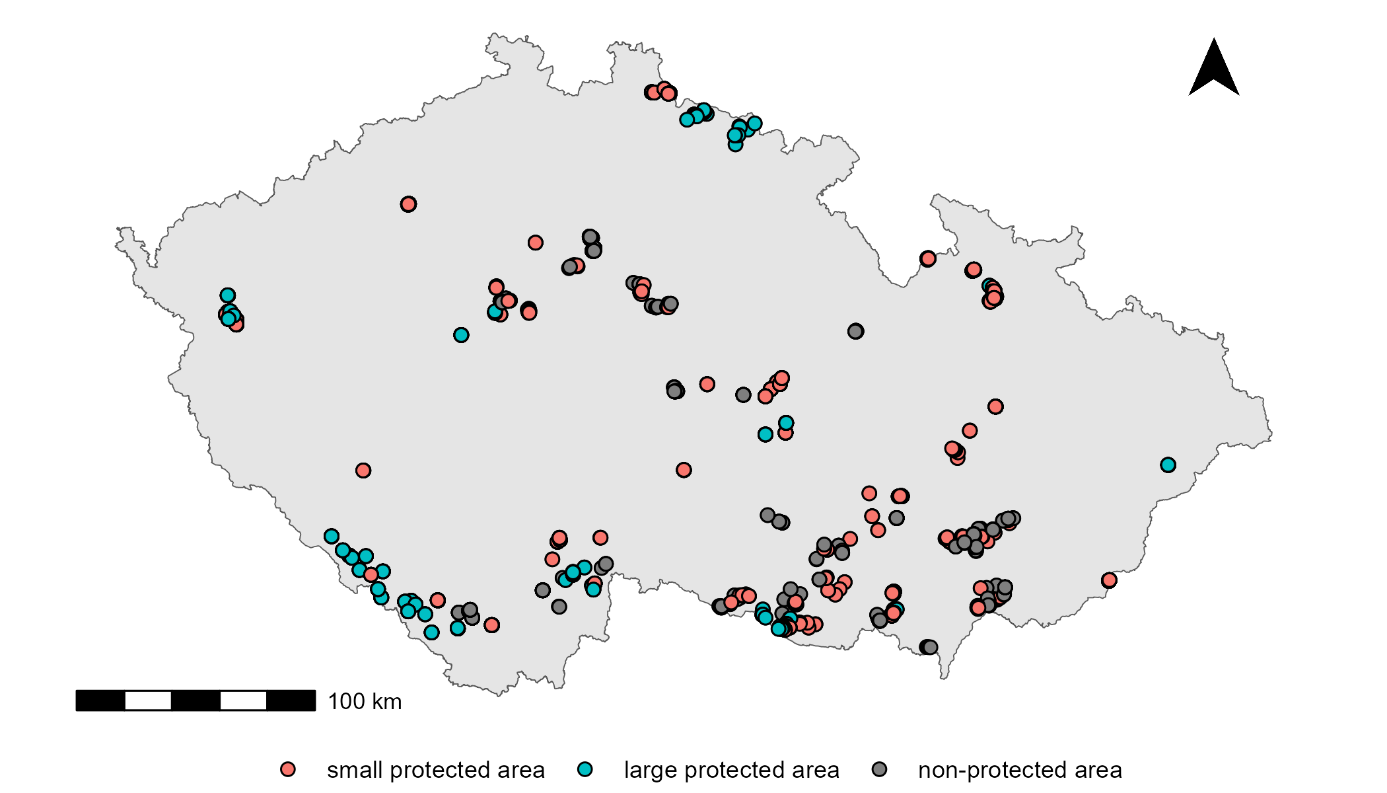


*Figure S2.7: Geographic distribution of repeatedly sampled vegetation plots. Plots in small and large protected areas and outside of them are shown in different colours.*


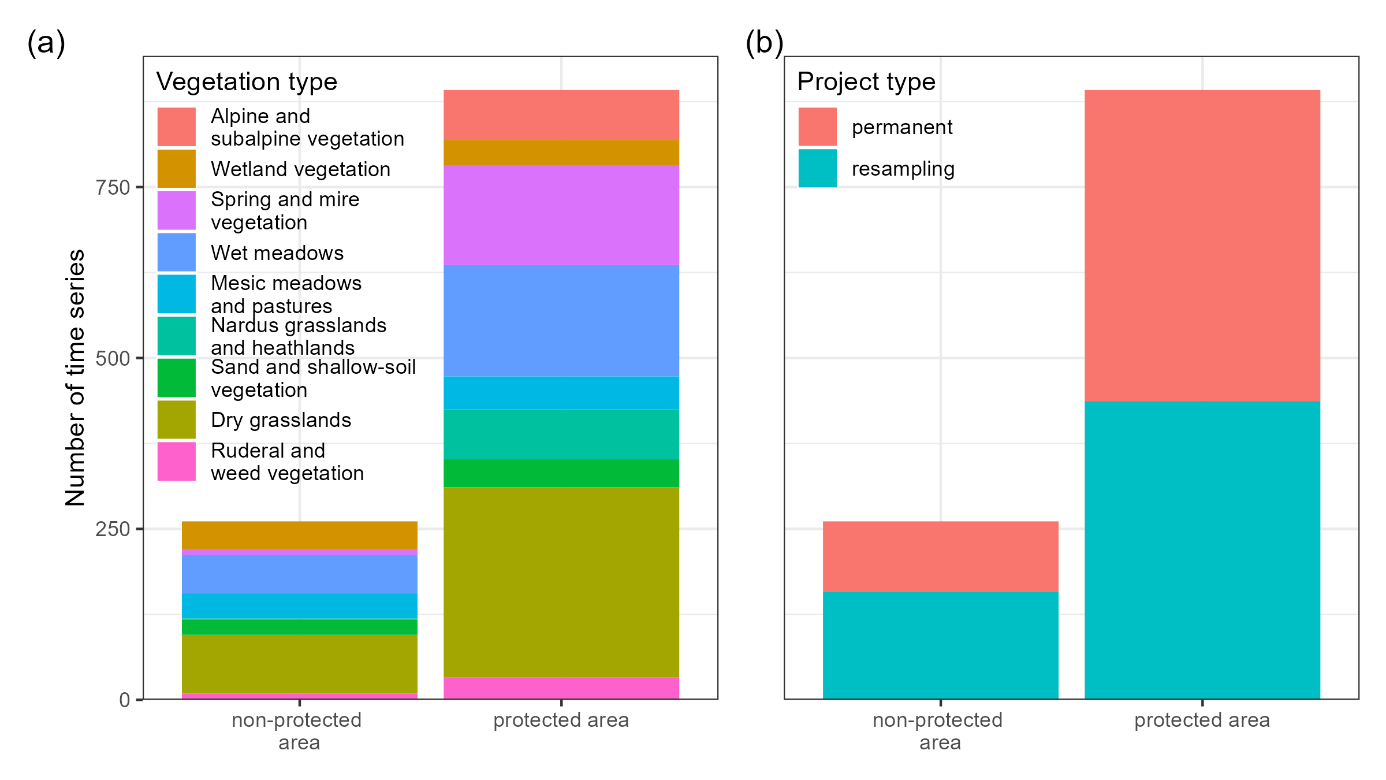


*Figure S2.8: Number of vegetation-plot time series in protected areas and outside of them. (a) Vegetation plots in different habitats and (b) permanent and resurveyed plots are shown in different colours.*


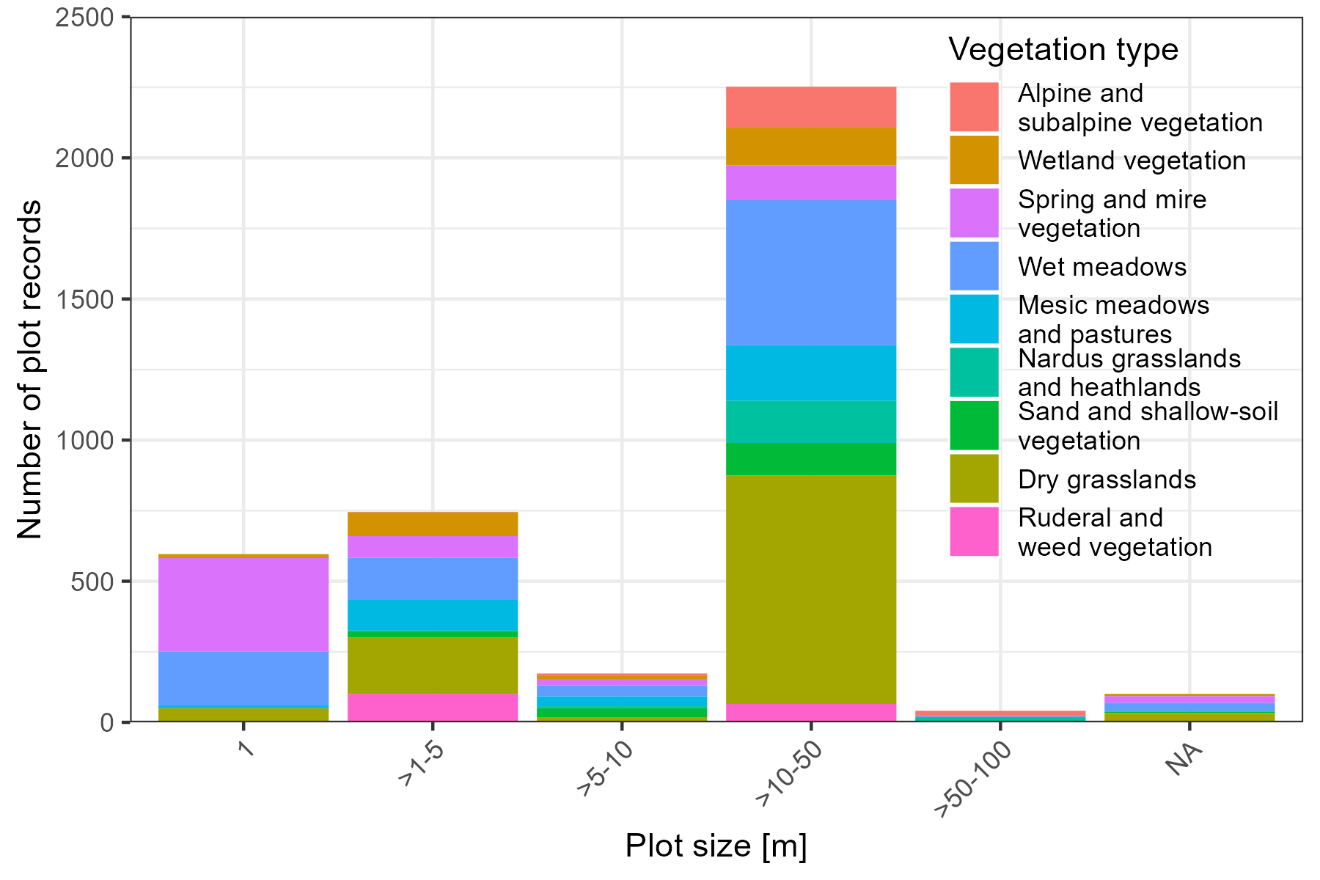


*Figure S2.9: Plot size distribution, plot records in different habitats are shown in different colours.*


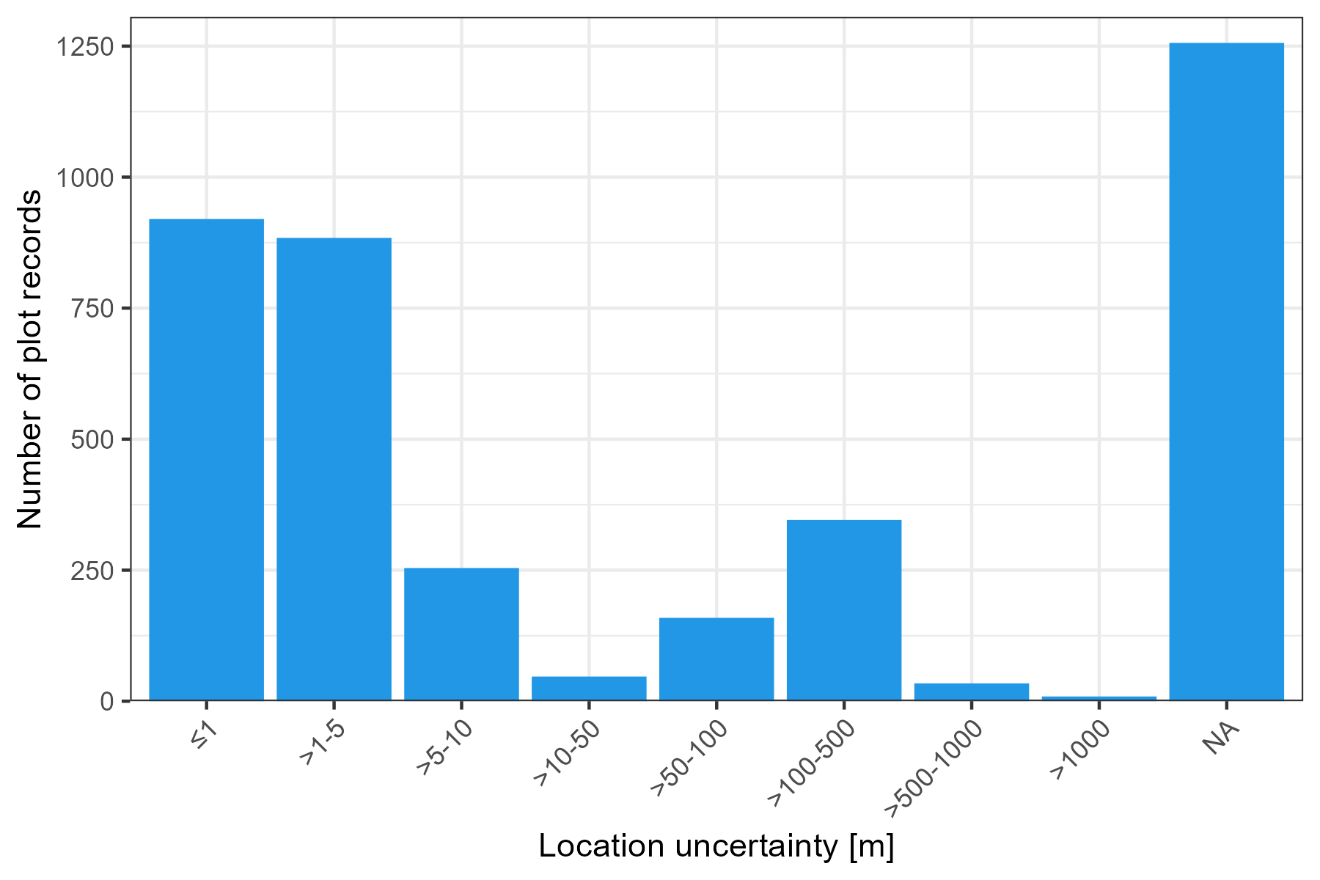


*Figure S2.10: Plot location uncertainty.*
