# Supplementary material for: Half a Century of Temperate Non‐Forest Vegetation Changes: No Net Loss in Species Richness, but Considerable Shifts in Taxonomic and Functional Composition
Source: Glob Chang Biol. 2025 Jan 24;31(1):e70030. doi: 10.1111/gcb.70030 (PMC11758476; doi:10.1111/gcb.70030)
Supplement: Supplementary file 4 — Appendix S6. [file GCB-31-e70030-s002.docx]

**Supplementary information to the article:**

Klinkovská et al. Half a century of temperate non-forest vegetation changes: no net loss in species richness, but considerable shifts in taxonomic and functional composition.

**Appendix S6:** Interval change approach to the analysis of changes in vegetation-plot time series.

We compared the results of two approaches to test changes in vegetation-plot time series data. In addition to the linear trend approach described in the main article, we used an interval change approach consisting of dividing each time series into separate observations of change between two consecutive surveys, following Jandt, Bruelheide, Jansen, et al. (2022).

For each interval, we calculated the change in species richness and Pielou’s evenness as log response ratios of the metric at time t_2_ divided by that at time t_1_. For the calculation of changes in species richness, we excluded observations of change between plots of different sizes. For the calculation of changes in Pielou’s evenness, we excluded observations of change between plots that differed in their size proportionally more than twice. In addition, we calculated changes in species characteristics as the difference between the community-weighted or unweighted means of the given characteristic at time t_2_ – that at time t_1_.

Similarly to the linear trend approach, we tested the probability of detecting a positive trend using a generalized additive model with a restricted maximum likelihood method (REML), assuming a binomial error distribution (positive trend = 1, negative trend = 0). We included spatial coordinates as a smoothing term based on spherical splines to account for spatial autocorrelation and the resurvey study identity (a group of related time series) as a random effect in the model to account for methodological differences between the different resurvey studies. We calculated the results for the whole dataset and each habitat type separately by including habitat identity as a predictor.

Most of the trends detected by the interval change approach had the same direction as those identified by the linear trend approach, although the proportion of significant changes detected by the linear trend approach was slightly higher (Fig. 1, Appendix S7).


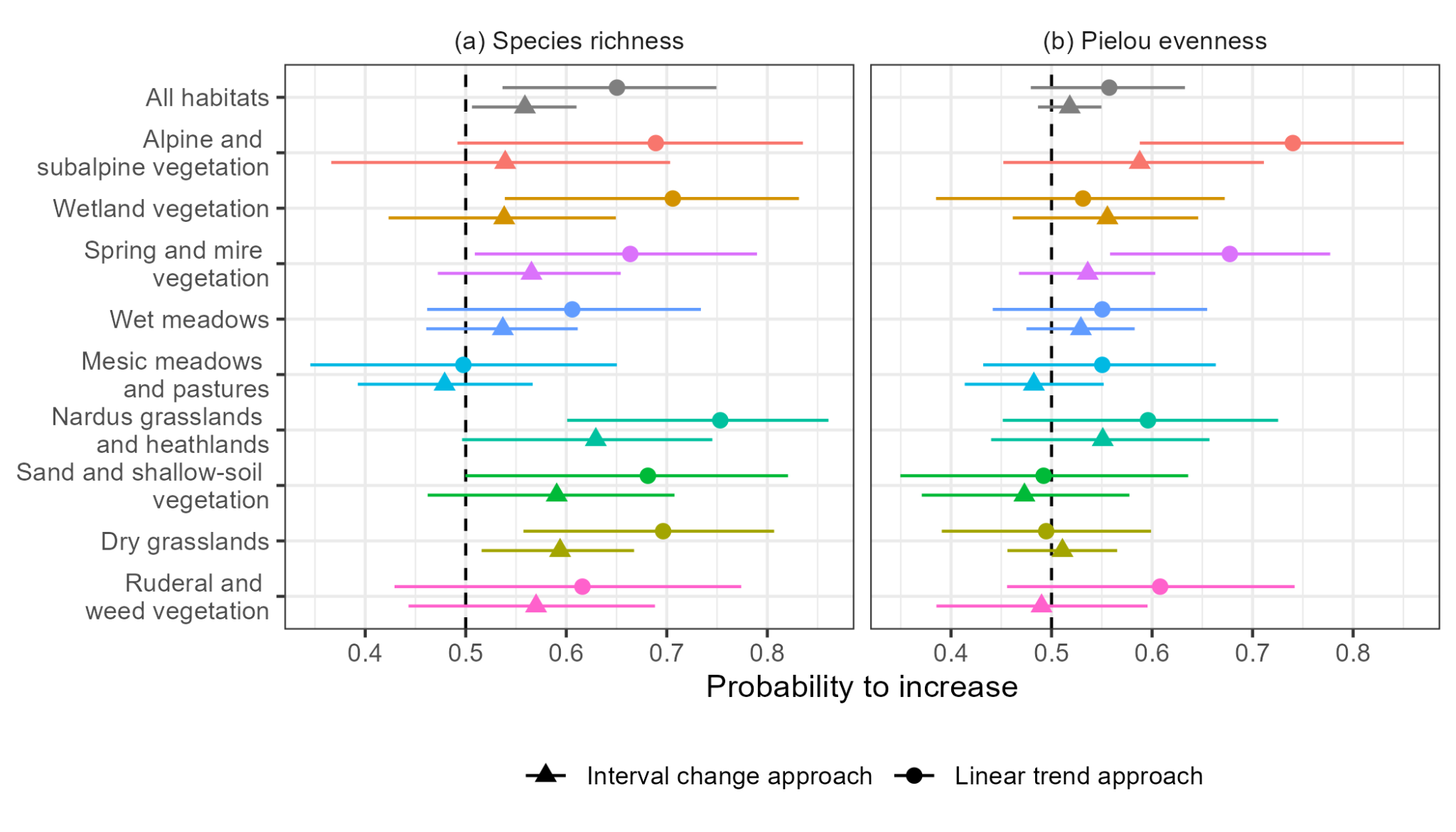


*Figure S6.1: The probability of detecting a positive trend for (a) species richness and (b) Pielou’s evenness in each habitat using the interval change approach and the linear trend approach within each time series. Points represent the probability estimates from the generalized additive models, and lines represent the 95% confidence intervals.*


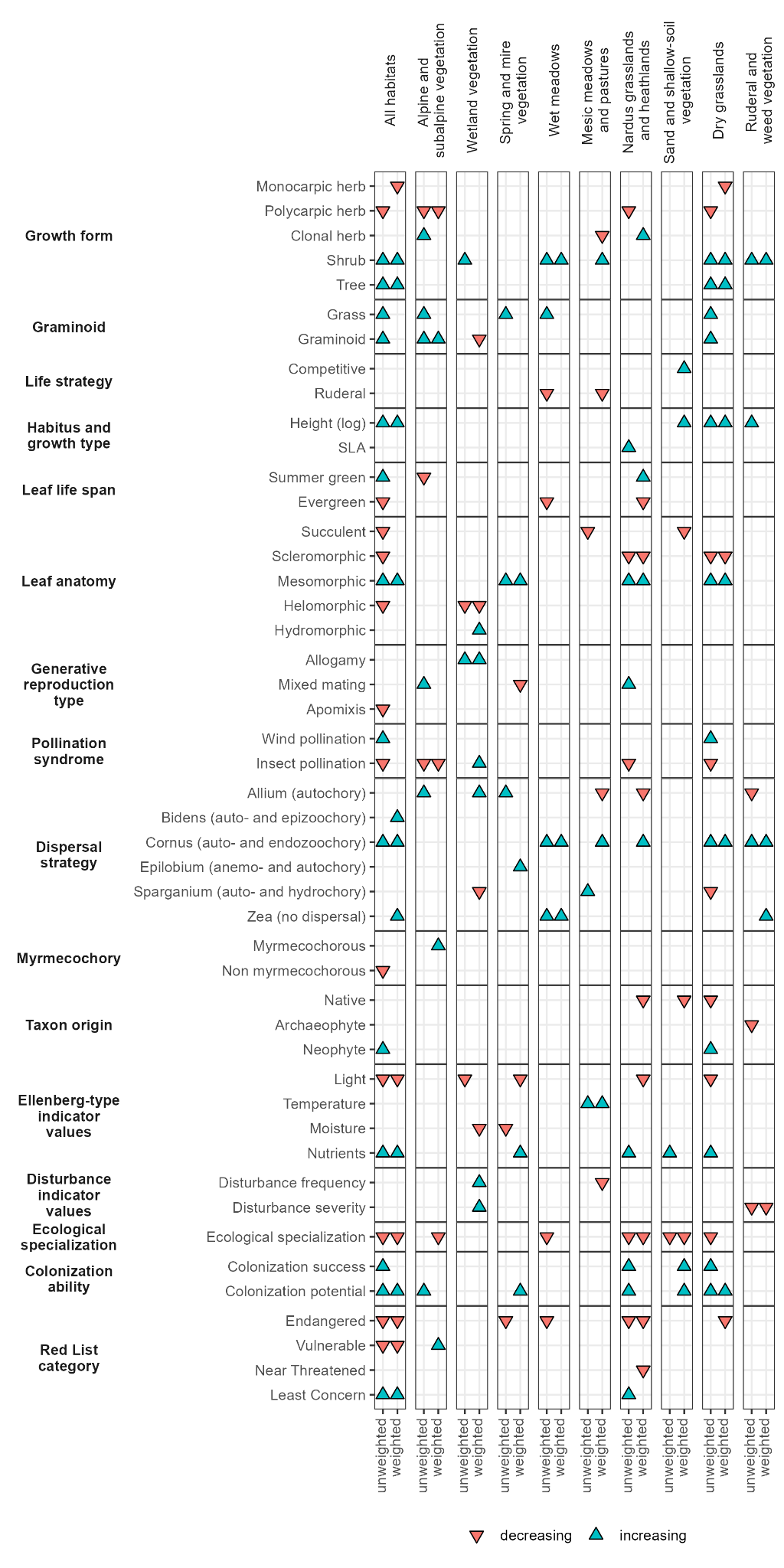


*Figure S6.2: Significant trends in community unweighted and cover-weighted means for each species characteristic and habitat identified using the interval change approach. Only species characteristics with at least one significant trend are shown.*
