# Supplementary material for: Half a Century of Temperate Non‐Forest Vegetation Changes: No Net Loss in Species Richness, but Considerable Shifts in Taxonomic and Functional Composition
Source: Glob Chang Biol. 2025 Jan 24;31(1):e70030. doi: 10.1111/gcb.70030 (PMC11758476; doi:10.1111/gcb.70030)
Supplement: Supplementary file 5 — Appendix S8. [file GCB-31-e70030-s003.docx]

**Supplementary information to the article:**

Klinkovská et al. Half a century of temperate non-forest vegetation changes: no net loss in species richness, but considerable shifts in taxonomic and functional composition.

**Appendix S7:** Species with significantly increasing and decreasing trends in the whole dataset and in each vegetation type.


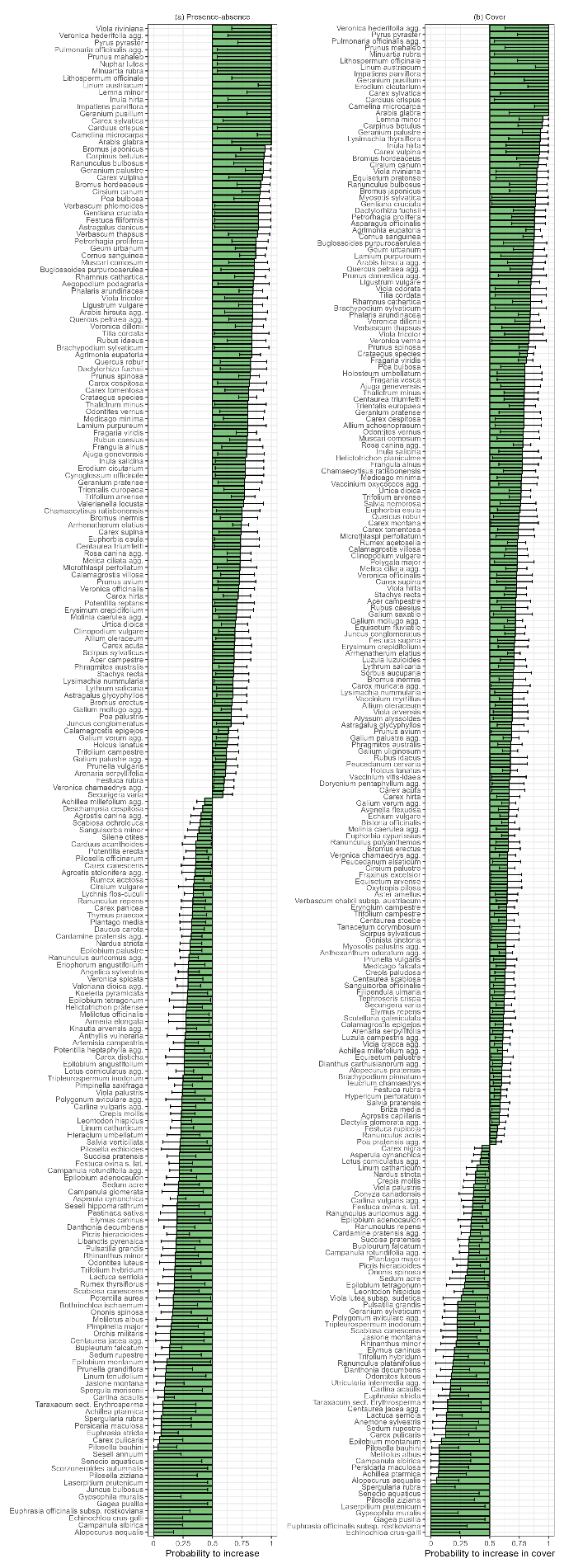


*Figure S7.1: The probability of an increase of a given species in its’ (a) presence and (b) cover in the whole dataset. The bars represent the estimated probability of increase from the binomial test and error bars indicate the 95% confidence intervals. Only species with significant trends are displayed.*

*
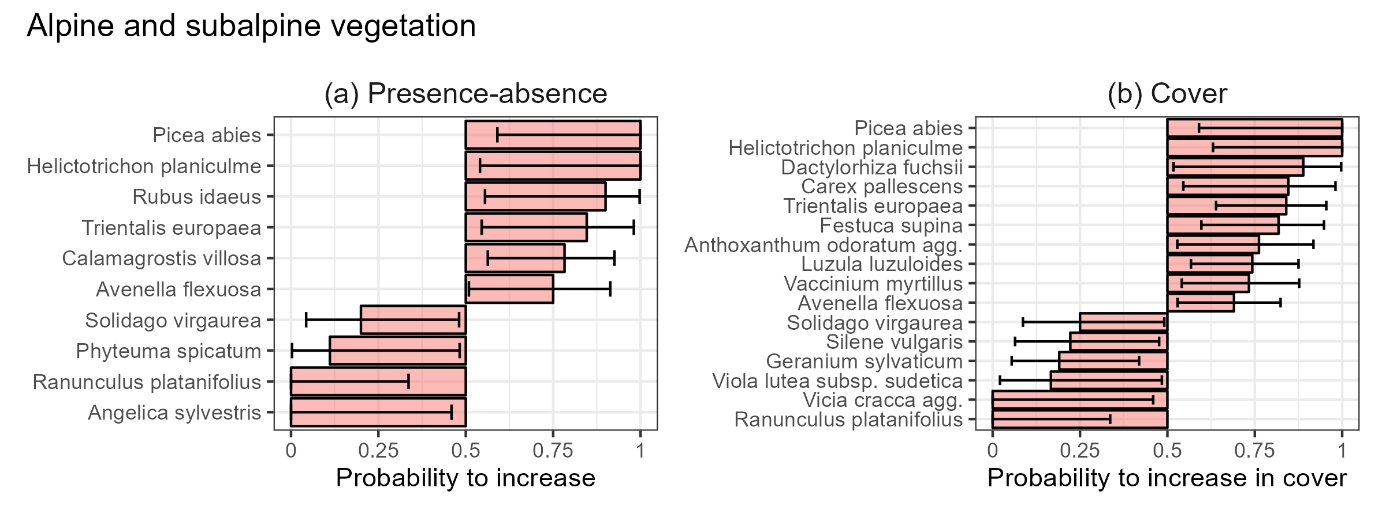
*

*Figure S7.2: The probability of an increase of a given species in its’ (a) presence and (b) cover in alpine and subalpine vegetation. The bars represent the estimated probability of increase from the binomial test and error bars indicate the 95% confidence intervals. Only species with significant trends are displayed.*


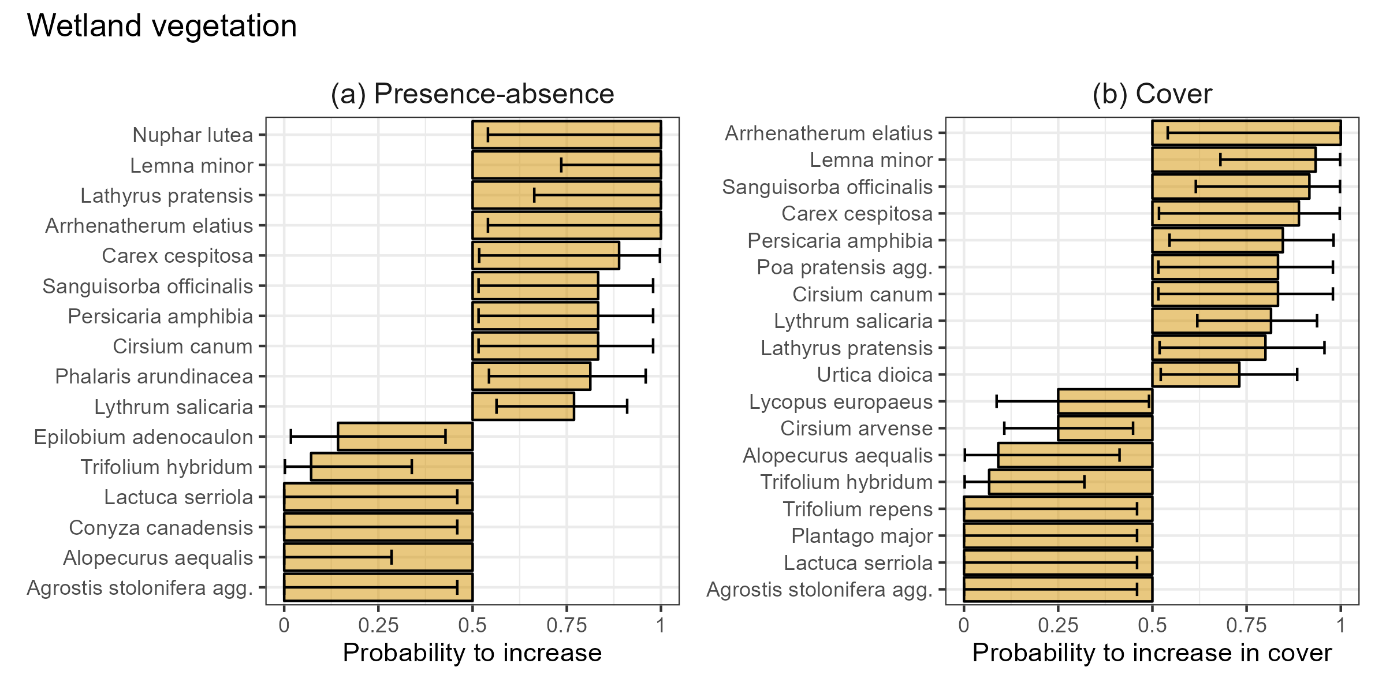


*Figure S7.3: The probability of an increase of a given species in its’ (a) presence and (b) cover in wetland vegetation. The bars represent the estimated probability of increase from the binomial test and error bars indicate the 95% confidence intervals. Only species with significant trends are displayed.*


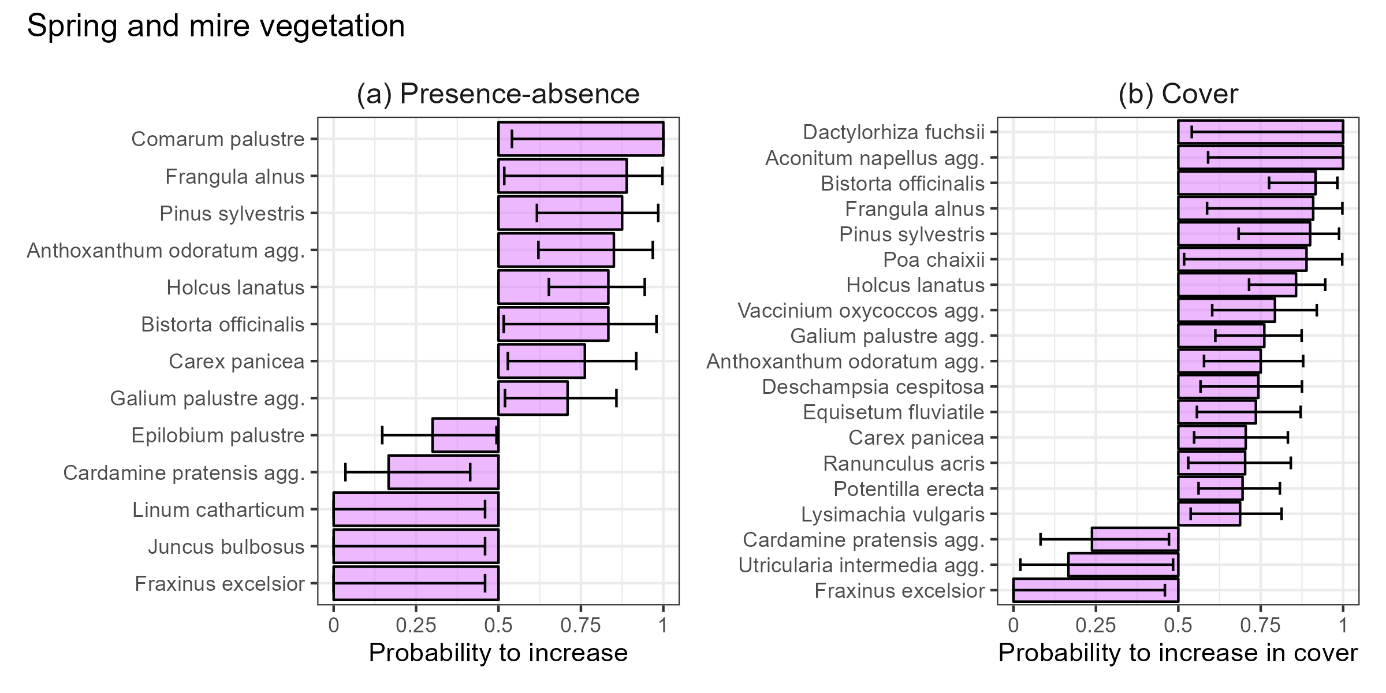


*Figure S7.4: The probability of an increase of a given species in its’ (a) presence and (b) cover in spring and mire vegetation. The bars represent the estimated probability of increase from the binomial test and error bars indicate the 95% confidence intervals. Only species with significant trends are displayed.*


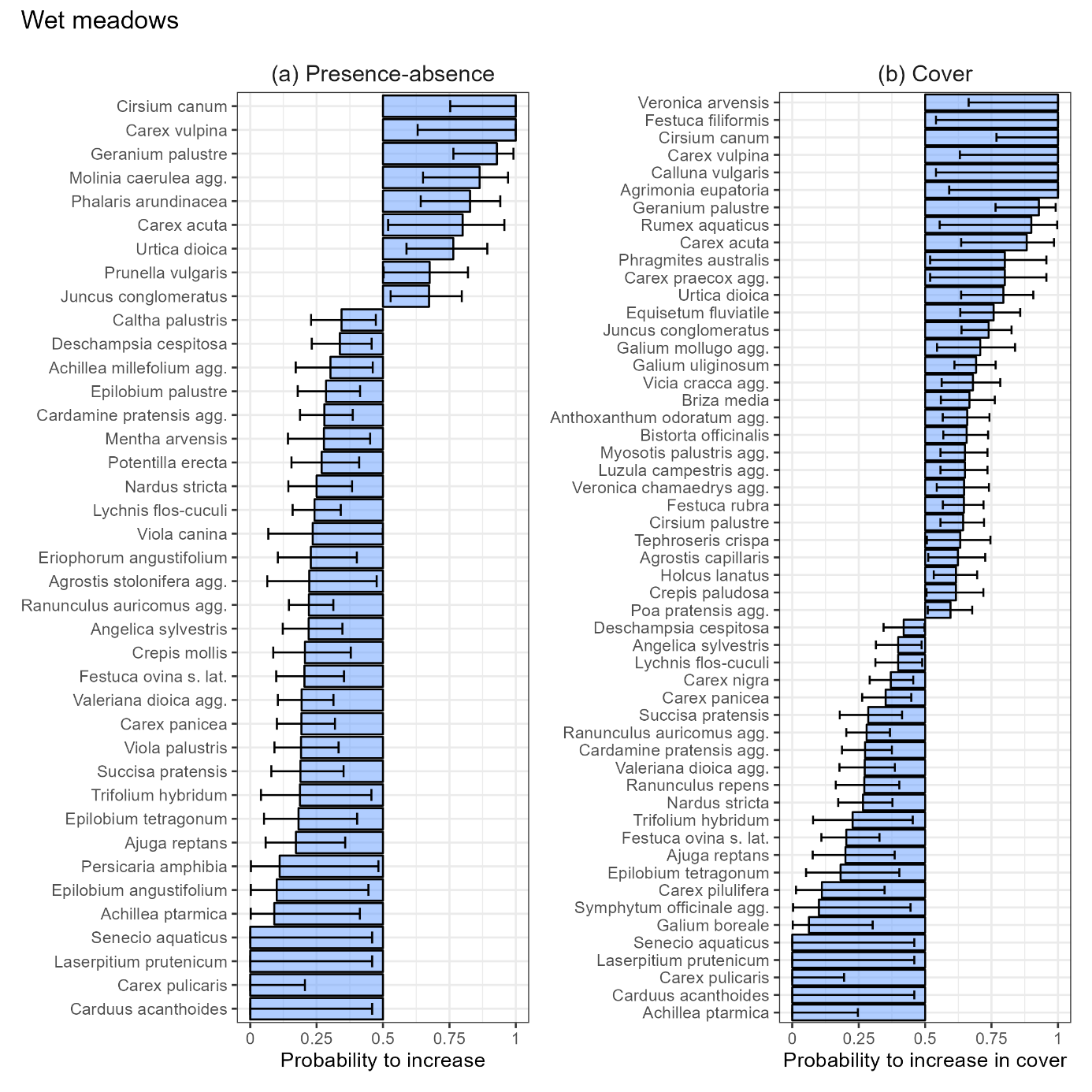


*Figure S7.5: The probability of an increase of a given species in its’ (a) presence and (b) cover in wet meadows. The bars represent the estimated probability of increase from the binomial test and error bars indicate the 95% confidence intervals. Only species with significant trends are displayed.*


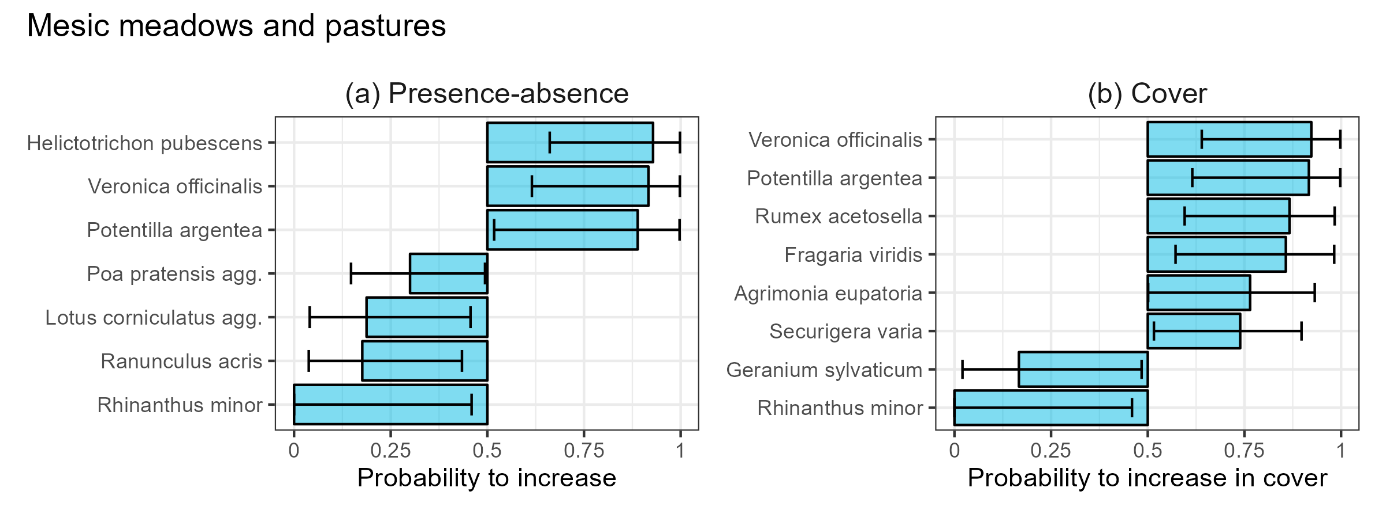


*Figure S7.6: The probability of an increase of a given species in its’ (a) presence and (b) cover in mesic meadows and pastures. The bars represent the estimated probability of increase from the binomial test and error bars indicate the 95% confidence intervals. Only species with significant trends are displayed.*


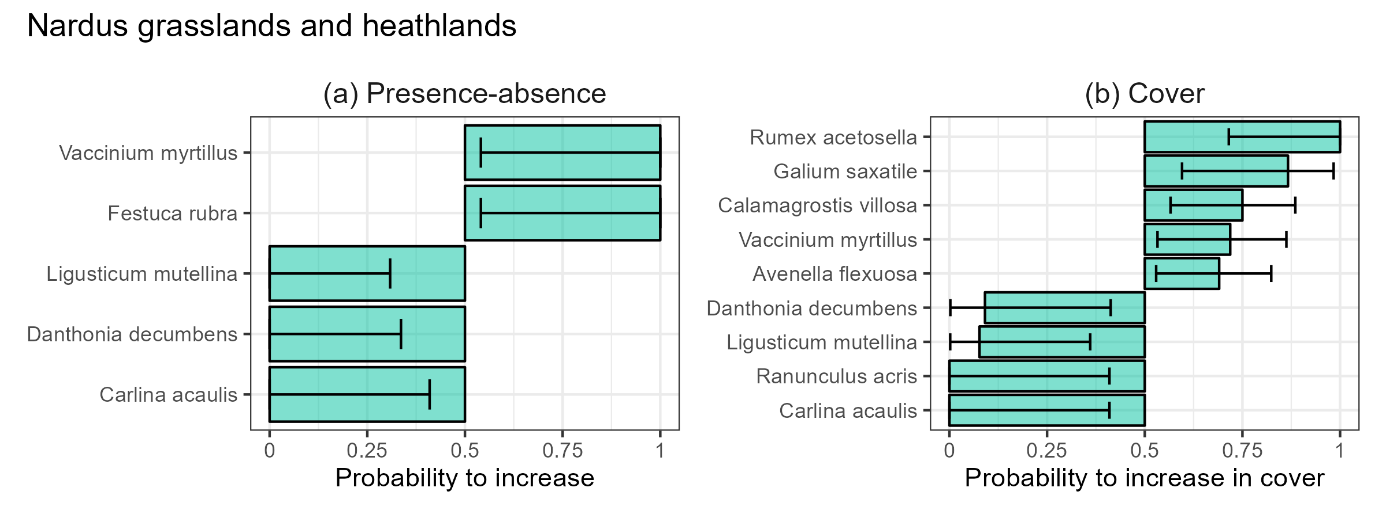


*Figure S7.7: The probability of an increase of a given species in its’ (a) presence and (b) cover in Nardus grasslands and heathlands. The bars represent the estimated probability of increase from the binomial test and error bars indicate the 95% confidence intervals. Only species with significant trends are displayed.*


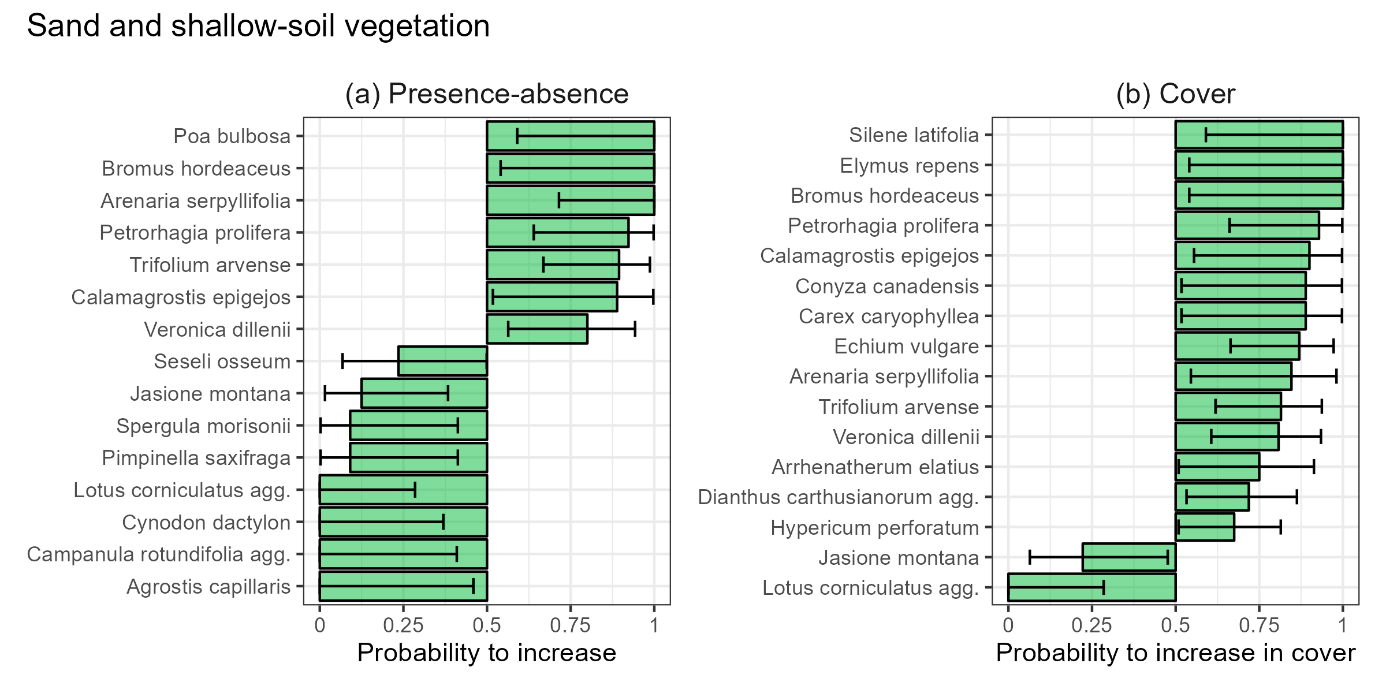


*Figure S7.8: The probability of an increase of a given species in its’ (a) presence and (b) cover in sand and shallow-soil vegetation. The bars represent the estimated probability of increase from the binomial test and error bars indicate the 95% confidence intervals. Only species with significant trends are displayed.*


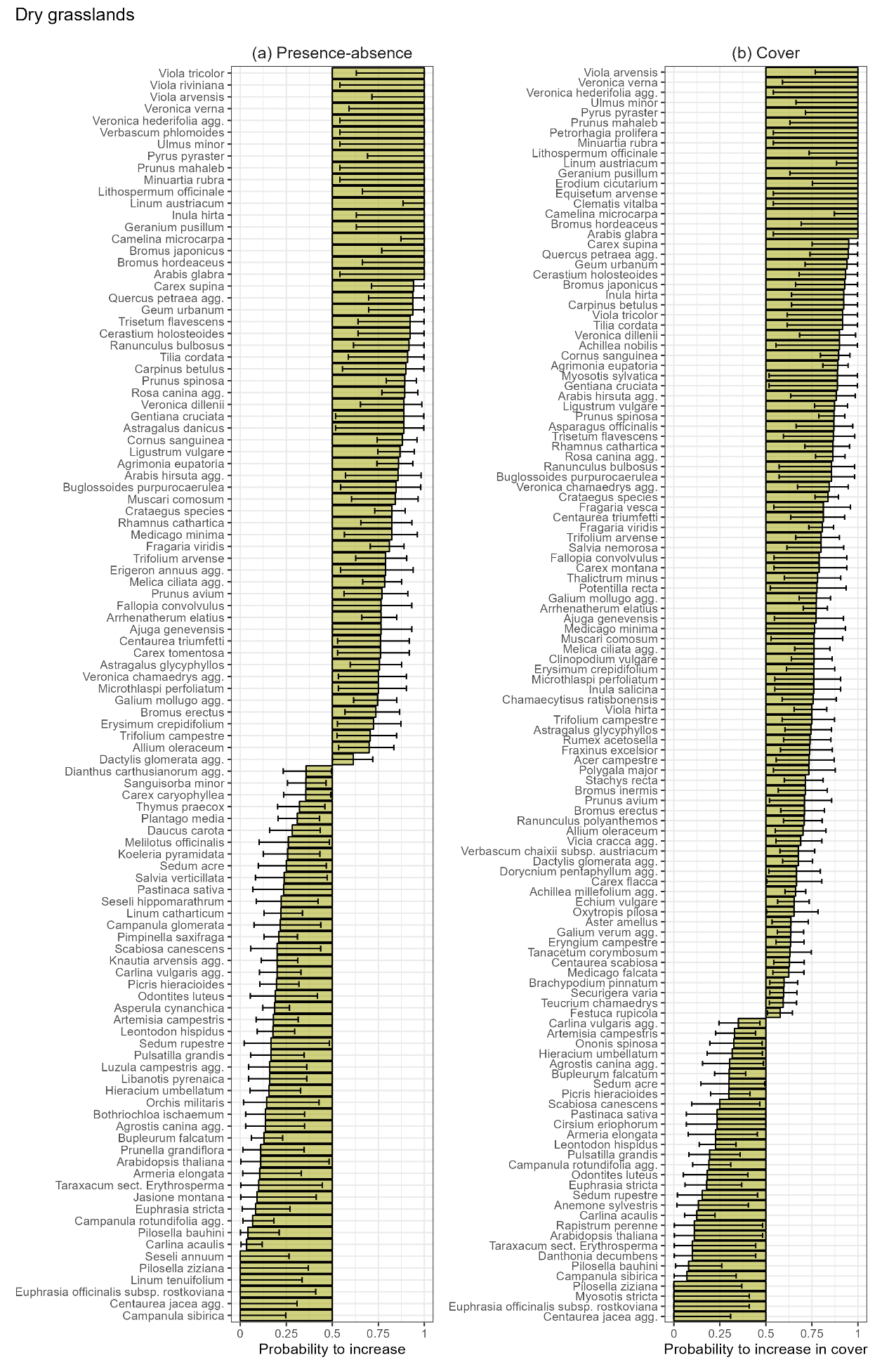


*Figure S7.9: The probability of an increase of a given species in its’ (a) presence and (b) cover in dry grasslands. The bars represent the estimated probability of increase from the binomial test and error bars indicate the 95% confidence intervals. Only species with significant trends are displayed.*


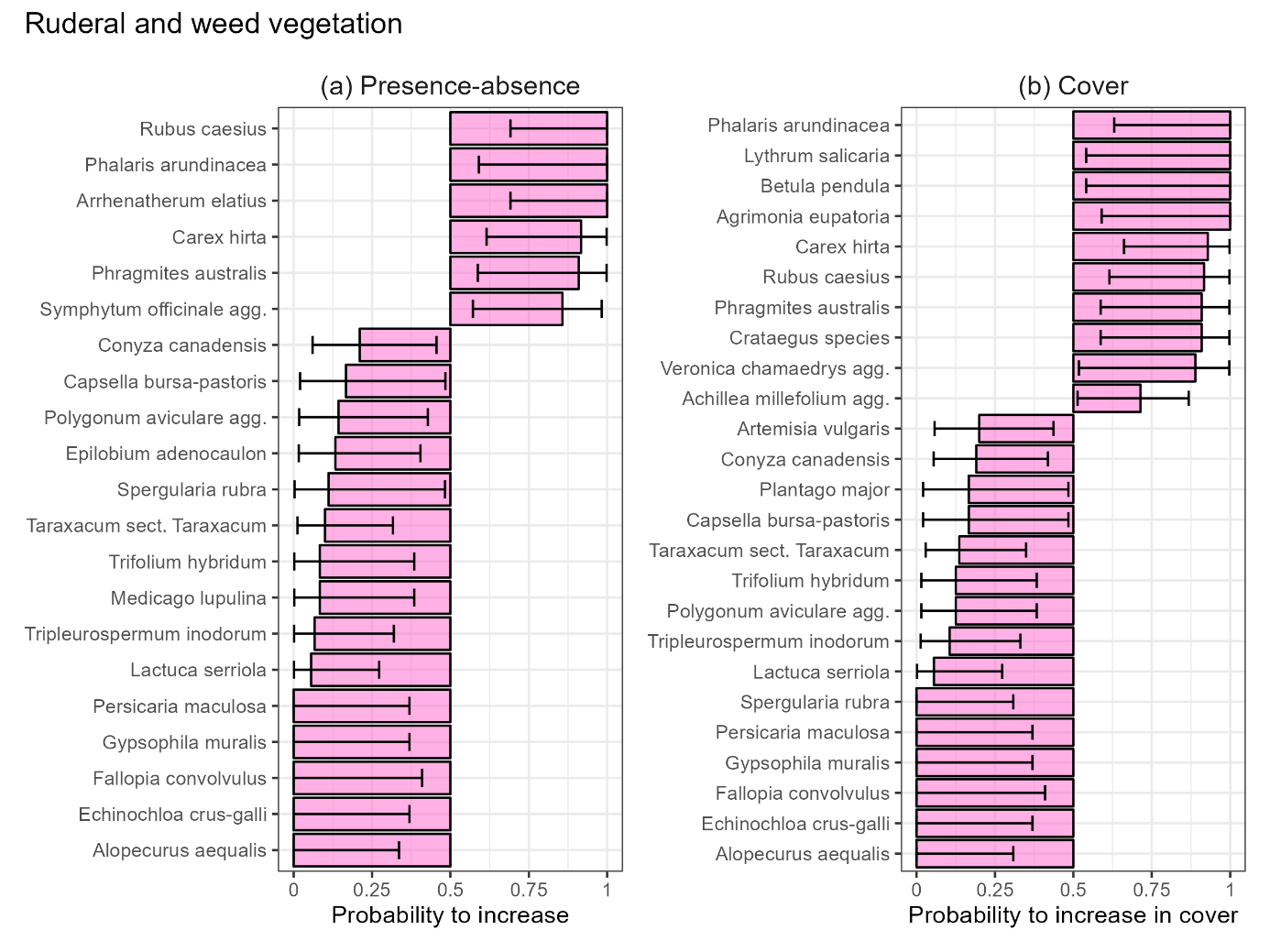


*Figure S7.10: The probability of an increase of a given species in its’ (a) presence and (b) cover in ruderal and weed vegetation. The bars represent the estimated probability of increase from the binomial test and error bars indicate the 95% confidence intervals. Only species with significant trends are displayed.*
